# Nanomaterial isolated extracellular vesicles enable high precision identification of tumor biomarkers for pancreatic cancer liquid biopsy

**DOI:** 10.1186/s12951-025-03527-3

**Published:** 2025-07-01

**Authors:** Zachary F. Greenberg, Samantha Ali, Andrew Brock, Jinmai Jiang, Thomas D. Schmittgen, Song Han, Steven J. Hughes, Kiley S. Graim, Mei He

**Affiliations:** 1 Department of Pharmaceutics, College of Pharmacy, University of Florida, Gainesville, FL 32610 USA; 2 Department of Surgery, College of Medicine, University of Florida, Gainesville, FL 32610 USA; 3 Department of Computer and Information Science and Engineering, Herbert Wertheim College of Engineering, University of Florida, Gainesville, FL 32610 USA

## Abstract

Pancreatic cancer has the highest mortality rate among all major cancers, which highlights the urgent needs of non-invasive early detection. Circulating extracellular vesicles (EVs) have gained significant attention for discovering tumor biomarkers. However, isolating EVs with well-defined homogeneous populations from complex biological samples is challenging. Different methods have been found to derive different EV populations carrying different biomolecules, which significantly confound biomarker discovery for developing clinical diagnostics. Building a rigorous EV isolation and standardizing assessment platform associated with -omics is essential to overcome this challenge. We introduced a novel isolation approach using a pH-responsive peptide conjugated with NanoPom magnetic beads (ExCy) for homogeneous EV isolation. Additionally, we introduced the first statistical algorithm for EV quality assessment (ExoQuality Index, EQI), which enables multi-assay quantification to provide a consistent and accurate definition of EV purity and quality; ExoQuality’s algorithm intakes multi-assay information to deconvolute complex EV heterogeneity. We performed the next generation sequencing on EV RNAs from pancreatic cancer patient plasma using four isolation methods; results highlighting ExCy’s isolation and EQI assessment improved biomarker identification. We identified a novel EV biomarker for pancreatic tumor, ATP6V0b, validated with quantitative PCR (qPCR) by screening a pilot cohort of 22 plasma samples. 16 were from pancreatic cancer patients, 6 with matched tumor tissue, and 6 healthy plasma samples. Through modelling the ATP6V0B cycling threshold, we reported 3 models with AUCs between 0.86 and 0.88, showcasing an enabling and clinically translatable liquid biopsy approach for early detection of pancreatic cancer using circulating EVs.

**Graphical Abstract:**

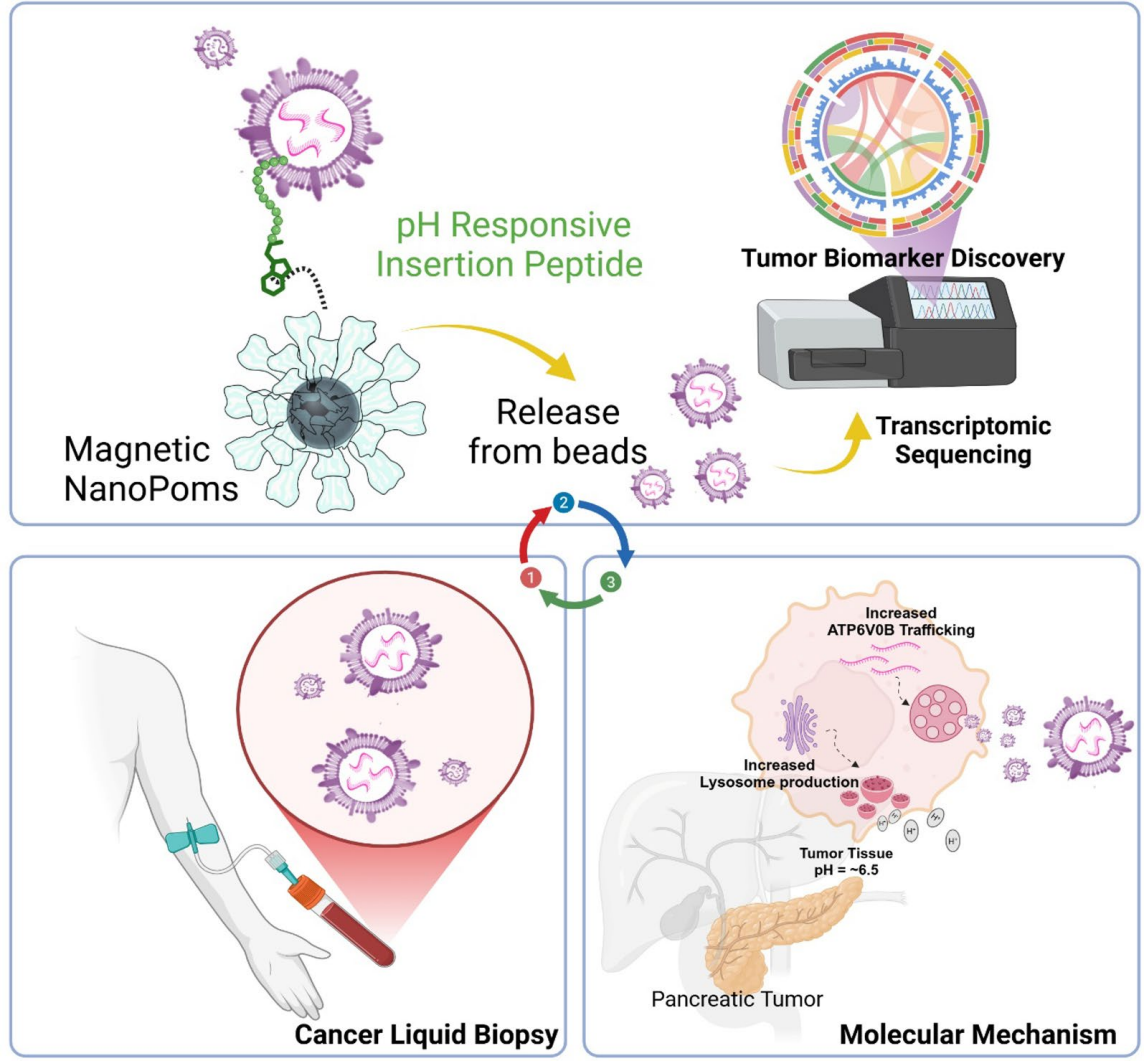

**Supplementary Information:**

The online version contains supplementary material available at 10.1186/s12951-025-03527-3.

## Introduction

Extracellular vesicles (EVs) have emerged as intriguing entities, garnering the attention of scientists from both basic and clinical research [1–3]. These small, membrane-bound nanovesicles hold immense potential for understanding diverse biological processes [4], such as intercellular communication and tumor initiation [5] and progression [6]. However, the isolation and purification of EVs present formidable challenges that profoundly impact downstream analysis and utility development with needed reliability and reproducibility [7, 8], particularly for handling complex biological samples. Biological fluids, such as blood [9], urine [10], cerebrospinal fluid [11], saliva [12], pleural effusion [13], ascites fluid [14], amniotic fluid [15], milk [16], bronchoalveolar lavage fluid [17], bacterial culture [18], and plant fluids [19], harbor an assortment of clinically relevant EVs. Current EV isolation techniques primarily rely on either size, density, or surface proteins, which lack ability to distinguish EV particles from non-EVs or diverse populations [20, 21]. Due to the complex heterogeneity of EVs, a single assay is unable to provide adequate data to define metrics of EV purity and quality. The lack of consistency has led to conflicting conclusions across studies [3, 7, 8, 20–35], which limits the clinical utility in routine diagnostic and therapeutic development. The Minimum Information for the Studies in Extracellular Vesicle research guidelines [20] (MISEV), published by the International Society of Extracellular Vesicles (ISEV) consortium, defines EV purity according to metrics including particles-to-protein and particles-to-RNA relative to the reference sample, and suggests using assays quantifying specific EV biomarkers in quantifying EV purity [20, 23, 24, 26–28, 36]. In consideration of non-EV contaminants, these MISEV’s metrics may be oversimplified as reported [7, 8, 25, 30–33]. There remains a significant gap in statistical metrics that enable rigorous analysis of EV isolates. We introduce an indexing strategy (ExoQuality Index, EQI) by leveraging data science to address this challenge of defining a rigorous, generalized statistical metric determined from an array of common EV analytical assays (e.g., nanoparticle tracking analysis (size and distribution) and total particle protein and RNA to evaluate EV isolate quality). We considered the sampling likelihood of each analytical assay across general EV isolation methods by applying a quantile–quantile transformation [37, 38] on our dataset, then demonstrated how EV isolation methods impact the EV quality by computing the EQI, a generalized EV sampling quality metric across methods.

**Fig. 1:**
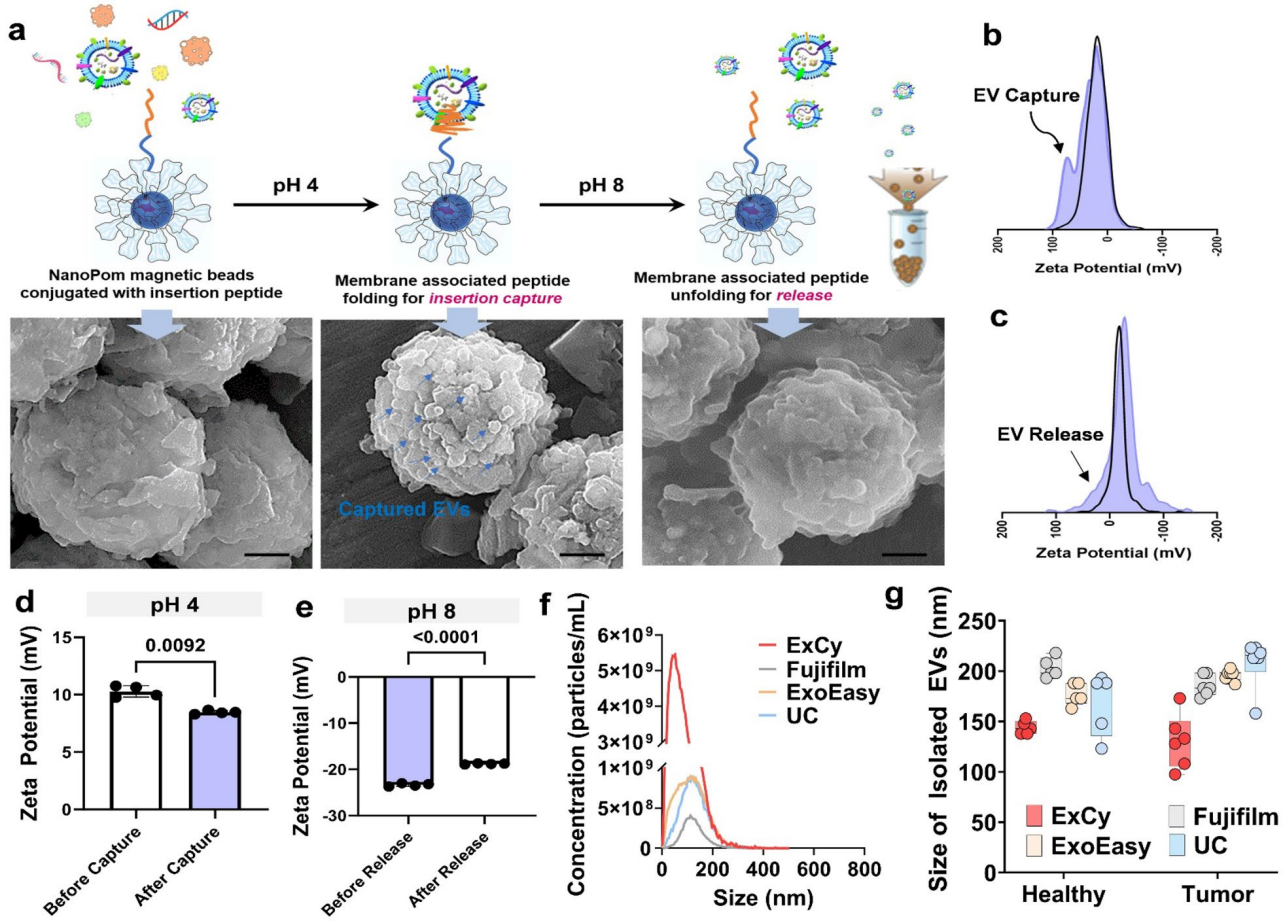
Capture-release ExCy magnetic NanoPom for EV purification from various biological fluids. **a** Schematic illustration of ExCy’s reversible capture-and-release of EVs with demonstration via SEM imaging. **b** ExCy’s zeta potential distribution profile during the capture and **c** release steps. **d** ExCy’s zeta potential before and after EV capture. n = 4 and CV ≤  ~ 3%. **e** ExCy’s zeta potential before and after EV release. n = 4 and CV ≤  ~ 3% **f** Nanoparticle tracking analysisn of 6 pateint samples compared across all methods (mean ± sd) in terms of particle concentration and size (**g**), indicating ExCy captures more small sized plasma EVs between 100–200 nm than ExoEasy, Fujifilm, and UC

We also introduce a novel capture-release EV isolation strategy (ExCy), which uses a pH-responsive peptide (pH-low insertion peptide, pHLIP) conjugated to our previously developed NanoPom magnetic bead surface [36]. The peptide crosses lipid bilayers to form an α-helix within the membrane for EV capture under acidic buffer conditions [39–41]. Unlike other reported peptide EV isolation approaches [42, 43], which use a biotin-linked irreversible insertion peptide, our EV isolation process is reversible upon restoring the buffer pH. Under the selected pH, EVs retained their original membrane integrity as proved previously [44, 45]. ExCy’s novel EV isolation is a simple, fast, and low-cost workflow that can be broadly employed to purify various biological fluids, including patient plasma and urines, cell culture medium, cow milk, bacterial culture, orange juice, and plant fluids (Fig. 1 and Additional file 1: Fig. S1). To demonstrate the EQI's generalization for evaluating EV isolation quality between isolation methods, we applied the EQI to compare isolated EVs from ExCy against EVs isolated from membrane affinity (ExoEasy Maxi Kit), sucrose cushion ultracentrifugation (UC), and phosphatidylserine affinity bead capture (Fujifilm MagCapture), using 6 pancreatic cancer patient plasma and 5 healthy control samples. For biomarker discovery, we selected 4 pancreatic cancer and 3 healthy plasma samples that retained high-quality RNA across the EV isolation methods. next, we conducted total EV RNA sequencing and transcriptomic analysis including differential analysis to seek tumor biomarkers across these methods. The HumanBase Pancreatic Network Enrichment analysis defined the ATP6V0B transcript as the statistically abundant gene from pancreatic patient plasma EVs using ExCy, but not from the other three isolation methods. We discovered that the ATP6V0B gene from patient plasma EVs could be a highly specific marker for diagnosing pancreatic cancer by validating a pilot cohort of 22 plasma samples (6 healthy, 16 patients including 6 matched tumor tissues). Using quantitative polymerase chain reaction (qPCR) to quantify the ATP6V0B gene from ExCy isolated plasma EVs, the average sixfold cross-validation AUC diagnostic value of 0.86–0.88 was achieved for using random forest [46], least absolute shrinkage and selection operatior (LASSO) [47], and linear discriminant analysis (LDA) [48] algorithms on ATP6V0b’s cycling threshold. ATP6V0B serves as a subunit of V-ATPase and mediates eukaryotic intracellular organelles, including protein sorting, zymogen activation, receptor-mediated endocytosis, and synaptic vesicle proton gradient generation (Human Protein Atlas) [49], which has been recognized as an essential oncogen in cancer progression by resected tumors and The Cancer Genome Atlas (TCGA) datasets [50]. Given that both V-ATPase and ATP6V0B are a part of the secretory granule pathway highly relevant to EVs origin, our results support the higher specificity and coverage range of our developed EV isolation method (ExCy) in terms of alignment with pancreatic tumor pathways and EV cellular component pathways. The majority of patients with pancreatic ductal adenocarcinoma (PDAC) present with locally advanced or distant metastatic disease (80–85%), and only a small amount of patients are surgically resectable (15–20%), which highlights the urgent needs for early detection of PDAC [51]. The discovered ATP6V0B may serve as a novel biomarker for early-detection of PDAC using circulating EVs, and broadly, as a hallmark to understand pancreatic tumor progression. Our introduced pipeline could be a generic platform for discovering novel tumor biomarkers from circulating EVs and enabling high-precision cancer liquid biopsy.

## Materials and methods

We have submitted all relevant data of our experiments to EV-TRACK (EV-TRACK ID: EV240005).

### Plasma collection and sample preparation

For the discovery cohort, the Clinical and Translational Science Institute, University of Florida, Gainesville, FL, collected blood and extracted plasma from 6 pancreatic cancer patients and 5 healthy individuals under the approved IRB protocol (IRB#202102401) with patient consent. The blood samples were collected using K2 EDTA tubes and plasma was extracted, then stored at − 80 °C for the study. Plasma samples were thawed from − 80 °C for processing. Sample processing and characterization were performed on the same day to avoid environmental change-associated variations. For the validation cohort, 22 plasma samples were collected using the approved IRB protocol (IRB#201600873). Of these 22 plasma samples, 16 were from pancreatic cancer patients, while 6 were from healthy individuals. In these 16 patient samples, 6 had matched tumor samples. As illustrated in Additional file 1: Fig. S1, two rounds of pre-clearing low-speed centrifugation were applied to remove cellular debris. Final volumes for all pre-cleared plasma samples used in this study were 1 mL. Additional file 1: Table S1 summarizes the differential epidemiological data including sex, age, pathology, histology, gender, ethnicity, race, and treatment status.

### ExCy bead fabrication and isolation

The pH-responsive peptide conjugated beads comprise our previously published unique nanographene pom pom-like microbeads (NanoPom) [36]. Briefly, the NanoPom is fabricated by using a Fe_3_O_4_/SiO_2_ core, followed by a series of sequential layer depositions of nanographene to form pom-like sheet layers with nanocavity in between, which enables a size hindrance to avoid considerably larger membrane structures. Streptavidin is cross-linked onto the beads, allowing a biotinylated insertion peptide conjugation to form ExCy beads. The pH-responsive peptide was synthesized through the KU Molecular Probe Core using a peptide microwave synthesizer and purified and analyzed by LC–MS. The quality and yield of synthesized peptides were characterized in Additional file 1: Fig. S2.

To capture EVs, 40 μl (approx. 0.4 mg beads) of ExCy was mixed with 1 mL of plasma. Depending on the original pH of biological sample fluids, the corresponding volume of HCl in 3 M concentration was added to the sample solution along with 5 uL of pH indicator Methyl Purple to achieve a buffer pH of around ~ 4. Detailed validation for various biological fluids was illustrated in Additional file 1: Fig. S1. Afterward, the sample mixture was incubated on a revolver for 1 h at 4 °C. Methyl purple was added to ensure the pH of the buffer between 4 and 6. After incubation, captured EVs were magnetically aggregated to remove the liquid solution for washing 3 times with ice-cold, pH 4, 1 × PBS. Subsequently, the washed beads solution in 40 μl was transferred to a 200 uL solution of pH 8, 1 × PBS containing 10 mM Tris for gentle vortex and to release for harvesting intact EVs. The detailed protocols, along with specific biological sample types, were shown in Additional file 1: Fig. S1.

### ExoEasy Maxi Kit for EV isolation

ExoEasy (QIAGEN) was used according to the manufacturer's protocol. 1 mL of plasma was added to an equal volume of XBP reagent, mixed, and centrifuged at 500× RCF for 5 min at room temperature. The membrane was washed with XWP reagent using 3000× RCF for 5 min at room temperature, followed by adding 400 uL of XE reagent for a 5-min centrifugation at 500× RCF at room temperature. We reapplied the eluate to the column, as per manufacturer recommendation, and centrifuged for 3000× RCF for 5 min to obtain EVs.

### Fujifilm MagCapture for EV isolation

Fujifilm’s MagCapture Exosome Isolation Kit PS v1 was used per the manufacturer’s protocol. 60 uL of magnetic beads were combined with 500 uL of Exosome Capture Immobilizing Buffer, mixed, and supernatant removed. 10 uL of Biotin-labeled Exosome Capture reagent was added to the beads in 500 uL of Exosome Capture Immobilizing Buffer for 10 min at 4 °C. The beads were washed 3 × with 500 uL of the Exosome Capture Immobilizing Buffer. Notably, 2 uL of Exosome Binding Enhancer was added to 1 mL of plasma sample. Washed beads were magnetically removed, applied to the plasma sample, and incubated for over 3 h at 4 °C. After incubation, 6 uL Exosome Binding Enhancer was added to 3 mL of Exosome Washing Buffer. This washing buffer was used to wash EV-captured beads 3×, followed by eluting twice with 50 uL of the Exosome Elution Buffer for a final eluate volume of EVs in 100 uL.

### Sucrose cushion ultracentrifugation (UC) [52]

A 3 mL of 30% (w/v) sucrose solution was placed at the bottom of an ultracentrifuge tube containing 15 mL 1 × ice-cold PBS. Next, 1 mL of plasma was gently added to the ultracentrifuge tube without disturbing the sucrose cushion, followed by a round of ultracentrifugation at 120,000 × RCF for 1.5 h at 4 °C. Next, the sucrose cushion was transferred to a different ultracentrifuge tube, 15 mL of 1 × ice cold PBS was added, followed by pipetting to gently break the sucrose cushion and ultracentrifuged again at 120,000 × RCF for 1.5 h at 4 °C. The supernatant was removed, followed by pellet resuspension in 200 uL 1 × PBS to collect EVs.

### Preparation for downstream analysis

After EV purification using four different isolation methods (ExCy, ExoEasy, FujiFilm, UC) from the same patient samples, all isolates received 25 mM PBS-trehalose [22] up to a final volume of 500 uL. Next, 300 uL was used for NTA, 18 uL for protein extraction, 5 uL for TEM, and 77 uL for RNA extraction. Each measurement was conducted 4 independent times with 4 replicates per measurement.

### Scanning electron microscopy

Field emission-scanning electron microscopy (FE-SEM) was used for visualizing the bead morphology, topology, and EV sizes, pre- and post-EV capture. Pre-capture beads were washed 3 × with ice-cold pH 4, 1 × PBS, and resuspended in 25 uL washing buffer. Post-capture beads were washed 3 × in ice-cold pH 8.0 1 × PBS with 10 mM Tris and resuspended in 25 uL of releasing washing buffer. Each resuspension solution was gently mixed and then directly aliquoted onto a 100% acetone-cleaned Ted Pella aluminum pin stub mount with complete solution evaporation. Next, the samples were sputter-coated for 30 s with an Au/Pd target using a Denton Desk V Sputter Coater and loaded into a Hitachi SU5000 Schottky Field-Emission Scanning Electron Microscope at a high negative vacuum pressure of 10–8 torr. An incident electron beam was applied to the samples at 7 keV and a beam current of 16.7 nA. Aperture and stigmata corrections were done before sample images were obtained.

### Transmission electron microscopy

Transmission electron microscopy (TEM, FEI Spirit TEM 120 kV) verified the morphology of isolated EVs. Briefly, ultrathin copper grids coated with 400 mesh carbon film (FCF400‐Cu‐UB, Electron Microscopy Science, USA) were used with glow discharge treatment for 1 min before use. Then, 5 μl EV samples were individually added onto the glow‐discharged grids and were quiescent for 10 min at room temperature. The grids were washed with distilled water once, then negatively stained with filtered 2% aqueous uranyl acetate for 2 min and dried at room temperature before observation. The TEM imaging power was 120 kV by FEI Spirit G2 with a digital camera (Soft Image System, Morada and Gatan Orius SC 1000B CCD‐camera).

### Nanoparticle tracking analysis

Nanoparticle tracking analysis was conducted using ZetaView (QUATT, Particle Metrix Inc, USA). ZetaView measures the nanoparticle’s Brownian motion using an incident laser to determine the corresponding size. The nanoparticle motion is then tracked by the detector and recorded over time: The incident laser wavelength was 488 nm^−1^ with sensitivity at 75 and shutter time at 163, over 90 s at the highest video resolution for all 11 positions.

### Zeta potential measurement

Zeta potential was measured using the Litesizer 500 (Anton Paar, Austria). The Litesizer 500 measures the zeta potential by flowing an electric current between two electrodes within the cuvette, measuring the interfacial charge of the solution at the particle’s surface and correlating this to the particle’s surface charge. ExCy beads under different conditions were dispersed within a 0.1 × PBS solution at pH 4 or 8 for measurement.

### Protein extraction, SDS-PAGE, and simple Western

18 uL of Isolated EVs were lysed with 2 uL of 1 × ice-cold RIPA buffer. Samples were incubated on ice for 15 min, with 30-s vortexing every 5 min. At the end of the incubation, the samples were sonicated for 15 s. Total protein was then quantified with the Pierce BCA Protein Assay Kit (Thermo Fisher, USA). Per the vendor’s protocol, reagents A and B were mixed at a 24:1 ratio to formulate the working buffer. In a separate tube, 10 uL of the working reagent and lysed EV sample were added, followed by incubation at 75 °C for 5 min, then cooled to room temperature to measure 562 nm^−1^ absorbance. Known quantities of bovine serum albumin were utilized to generate the standard calibration curve. For SDS-PAGE, 1 uL of Halt’s 100 × Protease Inhibitor Cocktail was supplemented to lysed samples. Approximately 20 ug of protein from each sample was resolved on a 4–20% gradient gel (Biotek), then visualized by SimplyBlue SafeStain. After total EV protein was quantified across the EV isolation methods, Simple Western was performed by loading each lane with 1 ug total protein extract and imaged according to the manufacturer’s procedure.

### RNA extraction and bioanalyzer

Total RNA was extracted using the miRNeasy Mini Kit per manufacturer instructions from EVs pre-processed with 5 uL of 1 × DNase I and RNase buffer. Isolated EVs were lysed with QIAzol and incubated for 5 min at room temperature. Next, chloroform was added to separate the lipids, mixed vigorously, then incubated at room temperature for 3 min, followed by 12,000 × RCF centrifugation for 15 min at 4° to allow phase separation. The aqueous phase was extracted into a separate tube, mixed with 100% ethanol, and then transferred to the RNeasy Mini column. The column was centrifuged at 8000 × RCF for 15 s at room temperature, followed by washing with 700 uL of reagent RWT and centrifuging at 8000 × RCF for 15 s at room temperature. Next, 500 uL of reagent RPE was added to the RNeasy column and centrifuged for 2 min at 8000 × RCF. We further dried the membrane by centrifuging an additional minute at full speed. Afterward, 50 uL of RNase-free water was added to the column to elute the RNA from the membrane using 8000 × RCF for 1 min. The eluate was applied to the membrane an additional time. Five uL of 1 × DNase I was added to remove any leftover DNA, followed by measuring the RNA quality through the 260/280 nm^−1^ ratio (BioTek Cytation 5). Each sample's RNA was then analyzed using Agilent's 2100 Bioanalyzer (Agilent Technologies) with the total RNA 6000 Pico Kit, according to the manufacturer's protocol.

To prepare total EV RNA for qPCR, total EV RNA was isolated from on-bead captured plasma or pancreatic tissue tumor EVs through an adapted miRNEasy protocol. For pancreatic tumor tissue EVs, 1x, ice-cold PBS was added to the resected pancreatic tumor tissue, then the solution was pipetted up-and-down 50 times, followed by placing the mixed solution onto a roller for 15 min at 4 °C. Afterward, the supernatant was removed and subjected to ExCy’s protocol after standard pre-clearing centrifugations. For on-bead digestion, all ExCy-captured EVs were washed 3 × with ice-cold, pH 4, 1 × PBS, followed by adding 1 mL of Qiazol, vortexing, and incubating for 10 min on ice. After incubating for 10 min on ice, this solution was centrifuged at 10,000 × RCF at 4 °C for 10 min, followed by aspirating only 700 uL into a separate tube for further processing by the miRNEasy protocol. A magnet was used to ensure all beads remained at the tube bottom before aspiration. 10 ug of Glycogen was added during the Qiazol/chloroform incubation step before standard processing in miRNEasy.

### Next generation sequencing

All extracted RNA samples, analyzed from the Bioanalyzer, for the inputs to NEBNext® Ultra RNA Library Prep Kit, were prepared as 2 ng/uL concentrations for cDNA preparation and adaptor ligation per manufacturer instructions. Briefly, 50 uL of sample was used to generate the first cDNA strand with the thermocycler settings of 25 °C, 42 °C, 70 °C, and 4 °C for 10, 15 × 2 min, and then held, respectively. The second cDNA reaction was immediately performed at 16 °C for 1 h with the heated lit at 40 °C. NEB’s SPIR magnetic beads purified double-stranded cDNA, then combined with the End Prep Reaction mix and enzyme, mixed, and incubated in a thermocycler at 20 °C, 65 °C, and 70 °C for 30 × 2 min, and held until the adapter ligation reaction. Adapter, ligation master mix, and enzymes were mixed with the samples on ice, followed by incubation in the thermocycler at 20 °C for 15 min. The USER enzyme was added to the samples, mixed, and incubated at 37 °C for 15 min with the heated lit set to 45 °C. Samples were purified with SPIR magnetic beads again. Adapter ligated DNA was subjected to PCR after adding the NEBNext® Multiplex Oligo 96 Unique Dual Index Primer Pairs, adding unique i7 and i5 forward and reverse primers to each sample. The Qubit Fluorometer quantified the constructed RNA library, and the Bioanalyzer checked the quality. Finally, all samples with the unique bar codes were pooled and sequenced on a Nova Seq 6000.

### Transcriptomic analysis

Transcriptomic analysis was conducted through the FREYA pipeline [53]. Reads were mapped to the hg38 genome with HISAT2 [54], followed by assembly quality control using FastQC, Trimmomatic [55], and GATK tools [56] Picard and SplitNCigarReads. Next, DEXSeq-Count [57] was used to generate the read counts for each transcript. Transcripts less than an average of 10 reads were filtered from the analysis. For exploratory analysis in Fig. 3, the representative early stage pancreatic cancer patient with pT1pN0 tumor was selected, having similar mRNA transcript counts between the isolation methods. Differential expression testing was conducted by edgeR [58], which uses a negative binomial distribution for the generalized linear model explaining EV isolate differences while adjusting for each method’s contribution to the observed EV isolate’s mRNA. Within edgeR, samples were normalized using the weighted trimmed mean of M-values [59] Transcripts passing the false discovery rate cutoff were used in downstream enrichment analysis. Enrichment analysis of statistically significant transcripts was performed using gProfiler [60] and the HumanBase [61] community detection algorithm to identify tissue-specific functional network interactions enriched with differentially expressed transcripts. HumanBase builds genome-scale functional map of human tissues to serve as a profiler of gene-specific function within tissue networks. HumanBase will attempt to profile input genes with any interacting partners, illustrated as the interaction confidence.

### ExoView R100

The ExoView R100 was utilized for EV tetraspanin (CD9, CD63, and CD81) abundance levels per standard protocols provided by NanoView Biosciences (Now Unchained Labs). After NTA evaluation of particles isolated by each of the methods, all inputs to the ExoView R100 were standardized to the lowest particle concentration by using solution A (pH 7.4), then all samples were diluted to the manufacturer’s recommendation for particles obtained from plasma. Next, 40 uL of diluted sample was dropped onto the microarray chip, incubated overnight, and the assay was conducted as per manufacturer’s instructions.

### Quantitative polymerase chain reaction (qPCR)

Purified RNA from the adapted miRNEasy protocol was further cleaned and concentrated into 10 uL through Zymo’s RNA concentrator kit. 2 uL was used for the RiboGreen assay to measure the total RNA concentration before reverse transcriptase. We utilized the SuperScript™ IV VILO™ with ezDNase protocol per manufacturer’s instruction to prepare the purified RNA for reverse transcriptase. The thermocycler settings used were 25 °C, 55 °C, 85 °C, and 4 °C at 10 min, 60 min, 5 min, then held, respectively. Before reverse transcriptase, all inputs were standardized to the lowest RNA concentration, as determined by RiboGreen, followed by input into qPCR for detection of ATP6V0B and EEF1A1 with TaqMan probes in 20 uL reactions.

### Machine learning models

LDA, LASSO, and random forest models were utilized to determine *ATP6V0b’s* importance as a PDAC predictor for 22 total datapoints using the Ct values. 16 datapoints corresponded to pancreatic cancer patients and 6 to healthy individuals. We first performed a grid search under a StratifiedKFold [62] (n = 6 folds) cross-validation scheme to determine the best parameter set for LASSO and random forest. LASSO’s best parameter was C = 0.1, and the parameter set for random forest was 50 trees with no max depth, 1 sample per leaf and 2 per node. Afterward, each model using their best parameters was fitted to the dataset using the StratifiedKFold cross-validation scheme to determine balanced accuracy, sensitivity, specificity, precision, AUROC, and F1.

### Statistics

A two-tailed *t* test was conducted to determine ExCy’s capture of EVs by zeta potential in Fig. 1. All plots with error bars mean ± standard deviation (N = 4 per sample). Analyses and plotting were performed with Python v3.8, R v4.1 and GraphPad v9.0. The false discovery rate for statistically different transcripts was calculated by Benjamini-Hochberg [63].

## Results

### The pH enabled magnetic capture-release for specifically purifying EVs

Building upon our previously published NanoPom [36] magnetic beads, we introduced the surface conjugation of pH responsive peptides (Additional file 1: Fig. S2), which confers high surface area and nanocavities favorable to capture small EVs, in isolating a well-defined EV population (ExCy). The pH responsive peptides spontaneously translocate across the lipid bilayer and form an α-helix within the membrane for insertion and stabilization under acidic buffer conditions [39–41, 64–67], while alkalizing to release at pH 8. We validated EV isolation performance from ExCy beads using SEM (Fig. 1a), demonstrating effective EV capture and release visualized on ExCy bead surface before and after pH change. Since ExCy-captured EVs undergo multiple rounds of washing to remove non-EV debris before EV release, the EV population’s homogeneity remains high. We also measured the zeta potential profile for associated changes before and after EV isolation, as shown in Fig. 1b–e, distinctive profiles implicating EV capture and release individually. The zeta potential profile supports the observed surface property change due to EV capture, which leads to a second peak observed from the expanded Debye length by EV attachment (Fig. 1b), rather than a singular broad peak often observed with plasma protein debris in colloidal systems [68, 69]. For EV release, we also observed a significant decrease in peak width (Fig. 1c). The observed zeta potential profiles of EV isolation are consistent with our SEM observations. When compared to conventional isolation methods on pancreatic cancer patient plasma using nanoparticle tracking analysis (NTA), ExCy’s EV isolation showed higher particle concentration and comparable size range (Fig. 1f, g). Additionally, we are showing broad applicability for EV isolation from various biological fluids, including human plasma, mammalian cell culture medium, cow milk, bacterial culture, orange juice, and hemp juice, as summarized in Additional file 1: Fig. S3. ExCy’s reusability was also assessed to show consistent EV isolation performance after four cycles of EV isolation, as demonstrated in Additional file 1: Fig. S4.

### The ExoQuality algorithm (EQI) for assessing EV isolation quality

**Fig. 2:**
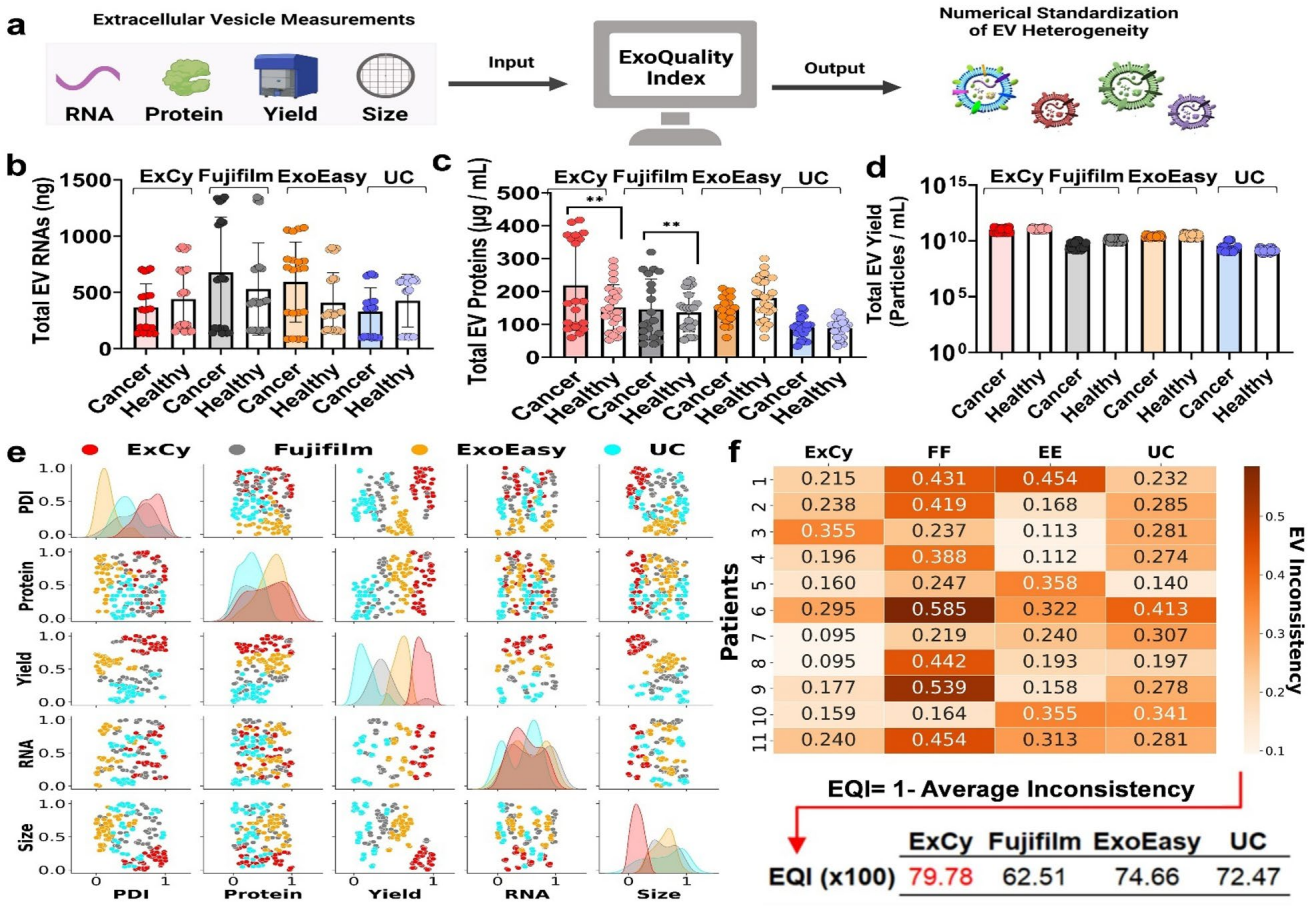
Statistical analysis using ExoQuality algorithm (EQI) to standardize EV isolation method for comparison. **a** Schematic illustration on how ExoQuality to evaluate quality of EV isolations based on multi-assay EV characterizations. **b** Stratification of patients to further investigate method-dependent purification of EVs in terms of total RNAs, **c** Proteins, and **d** EV particle yield. The 6 pancreatic cancer patients and 5 healthy control plasma samples were used. Four replicates for each sample. **e** Scatter plot matrix comparing the association of each EV measurement across the isolation methods. The particle dispersity index (PDI) [70] is a representation of the EV size distribution within a sample population, detailing observed heterogeneity. PDI was calculated as $\frac{\text{max}(NTA)}{{NTA}_{\sigma }}$ where NTA is the size profile and σ is the standard deviation. All isolations were done at the same date and location. The data was then transformed to a uniform distribution to adjust for each method’s bias contribution, applying a probabilistic rescaling within each EV measurement **f** Heatmap depicting the calculated EV Inconsistency value (EVI) across each method per patient. Within the probalistic rescaling, an EVI value of 0.5 represents a likelihood to observe 50% deviation of an EV isolate if resampled. **g** The ExoQuality Index metric used for direct comparison between EV isolation methods, interpreted as the expected likelihood of EV consistency, after resampling, to observe similar measurements

Due to the complex heterogeneity of EVs, a single assay is unable to provide consistent and accurate definition of EV purity and quality, which has been a significant challenge in translating the EV clinical utility. Presently, there is an urgency to develop standardization and rigorous cross-comparison EV isolation metrics. To address this gap, we introduced a generalizable metric for assessing EV isolation quality by employing a statistical algorithm to create an indexing strategy that intakes MISEV-suggested measurements [20], including total RNAs, proteins, yield, and size distribution. Multiple EV isolation methods including our developed ExCy, ExoEasy, Fujifilm, and UC, were used for validating EQI (Fig. 2a, Additional file 2). First, we applied each isolation method to purify 11 human plasma samples (6 pancreatic cancer patients and 5 healthy controls) to tabulate total RNAs (Fig. 2b), proteins (Fig. 2c), particle yield (Fig. 2d), size, and size distribution for analysis (Additional file 1: Fig. S5). The quality of isolated total EV RNAs and proteins from different isolation methods was further assessed by 260/280 nm^−1^ absorbance ratio (Additional file 1: Fig. S6) and SDS-PAGE gel electrophoresis (Additional file 1: Fig. S7).

We applied quantile transformation to each set of measurements (size, protein and RNA quantification, particle concentration, and particle dispersity index PDI) in our dataset, which standardizes data based on the cumulative distribution function for enabling direct comparisons across measurements in a probabilistic perspective [37, 38]. As seen previously [7, 8, 21, 25, 29–35], we observed distinct EV populations from each isolation method. The plots along the diagonal in Fig. 2e represent the univariate distribution of each EV quality measurement within an equal likelihood space, which indicates how each measurement is distinctive across the isolation methods. Our analysis shows that EV total RNAs and proteins may be EV isolation-independent metrics since their univariate distributions primarily overlapped. However, when we examined the univariate distributions for EV size, yield, and particle dispersity index, we observed that these EV metrics were heavily affected by their respective isolation methods, which may have been further affected by the technical bias imposed by nanoparticle tracking analysis. To address the analytical incongruencies for an isolated EV population across multiple assays and isolation methods, we computed the EV Inconsistency value (EVI) after quantile transformation to enable EV quality generalization. The EVI Eq. (1) is

1 $$EVI_{method} = \sum\limits_{i = 1}^{n} {\sqrt {Var[(i_{assays} )(replicates)]} }$$

where the aggregate standard deviation is calculated for all assays across all replicates for a given method; since we quantile transformed our data, this equation assumes that the assay variance observed is solely due to random error for the isolation method and not due to the underlying technical bias of the assay. Moreover, the aggregation of standard deviations will reflect the implicit inconsistency for a method isolating EVs. The EVI was tabulated across all patients and shown in Fig. 2f to compare the performance of all methods in between each individual patient. A value of 0.2 indicates that the expected EV population may differ by 20% after resampling within the same isolation method. For instance, in a pairwise sample comparison, Fujifilm may deviate more frequently than ExCy, ExoEasy, and UC. Therefore, we calculated each method’s expected consistency for EV isolation, interpreted as the *ExoQuality Index* = *1* − *Average(EVI)*, attempting to directly quantify comparisons between methods (Fig. 2g). The EQI 79.78% for ExCy indicates the highest likelihood to obtain consistent EV populations during isolation among all methods, suggesting homogenous EV population with less non-EV contamination. In order to validate the EQI metric, we also characterized the purified EV population using the ExoView platform [27] (Additional file 1: Figs. S8–S11) and Simple Western (Additional file 1: Fig. S12), in terms of contamination across methods by measuring plasma serum albumin and Apo-A1 lipoprotein. Through the ExoView Platform, we first observed that ExoEasy deposited a substantial amount of precipitate, leading to highly weak signal detection by the ExoView platform (Additional file 1: Fig. S8), while ExCy (Additional file 1: Fig. S9), Fujifilm (Additional file 1: Fig. S10), and UC showed co-localized EV tetraspanin signals (Additional file 1: Fig. S11), with Fujifilm showing the least co-localized EV signal. Simple Western revealed that ExCy and UC had no contamination by abundant plasma proteins including serum albumin and Apolipoprotein-A1 (ApoA1) while ExoEasy and Fujilfilm did show contamination (Additional file 1: Fig. S12).

### NGS for analysis of EV isolation quality at molecular precision

**Fig. 3:**
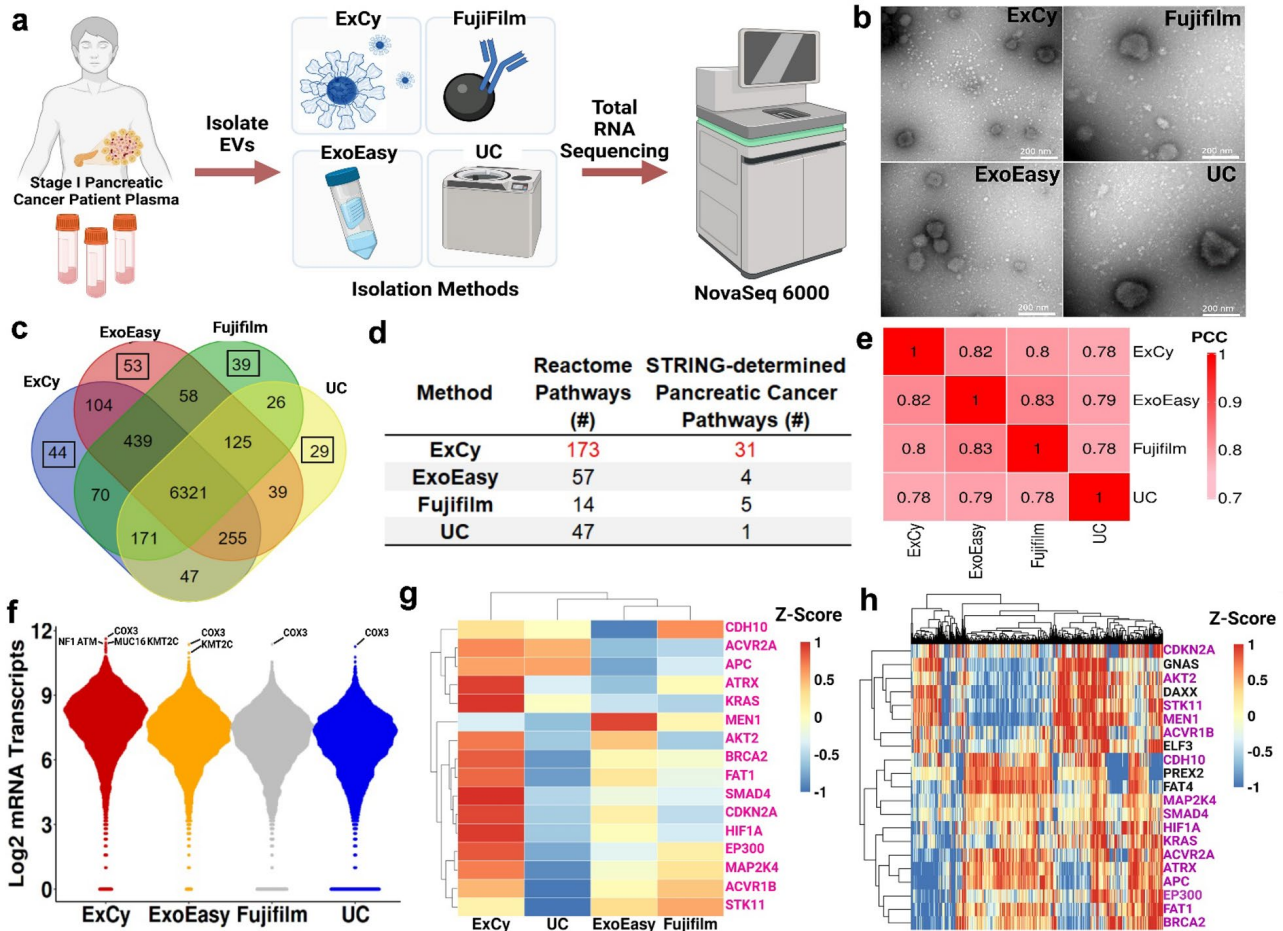
A patient zoomed-in exploratory analysis substantiates EV isolation quality. **a** Schematic Illustration depicting zoomed-in patient analysis. Stage 1 pancreatic cancer patient plasma in1 mL was used (Adenocarcinoma, pT1pN0, female, white). **b** Transmission electron microscopy showing EV morphology across the four isolation methods. **c** Venn diagram illustrates mRNA sequencing gene comparison between isolation methods. The highlighted box shows the number of unique mRNA annotations from different isolations. **d** Pathway analyses, using gProfiler and padj < 0.05, on the unique mRNA annotations across each method. STRING-associated pancreatic cancer pathways were obtained from determined Reactome pathways. **e** Method-by-method correlation matrix in mRNA profiles across methods. **f** Beeswarm plots depicting mRNA transcript distributions; top mRNA transcripts were annotated, followed by filtering for pancreatic cancer associations using TCGA’s Cancer Gene Consensus analysis. **g** COSMIC heatmap for all mRNA transcripts across each isolation method. Pink colored labels indicate isolated EV transcripts overlapped with TCGA database transcripts. **h** COSMIC heatmap using TCGA’s curated pancreatic cancer database to define patient baseline mRNA transcript level and profile to correlate with ExCy isolated EV transcript profiles. Pink colored labels indicate ExCy isolated EV transcripts overlapped with TCGA database transcripts

In order to precisely define purified EV populations, we used next generation sequencing (NGS) analysis to investigate the transcriptomic profiles from different EV isolates (Fig. 3). We quantified a total of 44 RNA samples using the Bioanalyzer (Additional file 1: Figs. S13–S16) to select early-stage pancreatic cancer patient (Pancreatic ductal adenocarcinoma, PDAC) plasma samples (n = 4) with healthy controls (n = 3) for EV purification using ExCy, ExoEasy, Fujifilm, and UC, and subsequent extraction of total EV RNAs, respectively. Utilizing the FREYA pipeline [53], we investigated the raw sequence assembly quality through FastQC (Additional file 2), followed by mapping to hg38 using HISAT2 [54]. HISAT2’s mapping rate across the methods indicated ExCy, on average, had the highest alignment rate and the least varied mapping rate (Additional file 1: Table S2), suggesting purer isolated EV RNA quality. Following this, we tabulated the RNA populations in each patient across all methods (Additional file 1: Table S3). Next, we quantified the method-dependent EV RNA annotations by querying to Vesiclepedia [71], to understand levels of non-EV reported annotations (Additional file 1: Table S4).Further increasing analytical rigor in the following analysis, we assessed RNA biotype distributions across all methods for standardizing referenced to Vesiclepedia. We noticed a large loss (4000–6000) in total annotated RNAs after standardizing to Vesiclepedia, and more strikingly, we observed significant variations in detected RNA biotypes across the methods for each patient, further implicating that observed RNA biotypes may be strongly biased by isolation methods.

Next, we performed exploratory analysis to assess per-patient mRNA differences across the EV isolation methods (Fig. 3, Additional file 1: Figs. S17–S19, Additional file 2)**.** We selected a representative tumor patient (Fig. 3a; stage I early pancreatic cancer) to show each method’s representative isolated EVs by TEM imaging (Fig. 3b) and average size quantification (Additional file 1: Fig. S21), indicating a clear disparity in particle size and distribution regarding the true EV population from this patient’s plasma. Next, we visualized annotated mRNAs comparisons across each method (Fig. 3c), followed by pathway analysis with gProfiler [60] on the unique mRNA annotations (Additional file 3) to probe whether the isolation-specific mRNA signatures indicate connections to known pancreatic cancer pathways. To do so, we tabulated all the statistically significant Reactome [72] pathways from gProfiler (Additional file 4), then cross-referenced against STRING [73] to find Reactome-associated pancreatic cancer pathways (Fig. 3d; Additional file 5). We statistically analyzed the Method-by-method correlation matrix for comparing similarity in mRNA profiles (Fig. 3e). Given the number of pancreatic cancer pathways found, we further investigated whether the methods were enriching for pancreatic cancer related markers, in which *COX3*, a prognostic marker in pancreatic cancer [49], appeared unilaterally (Fig. 3f). We applied TCGA’s Cancer Gene Census analysis, which queries transcripts against the Catalogue of Somatic Mutations in Cancer (COSMIC) [74]. The cancer-associated transcripts including *NF1*, *ATM*, *MUC16*, and *KMT2C*, were found at higher mRNA levels with ExCy (Fig. 3f). We also queried COSMIC [74] for pancreatic cancer related mRNA transcripts to visualize differences in their mRNA transcript levels between EV isolation methods (Fig. 3g). Notably, ExCy identified more cancer related mRNA transcripts compared to the other methods (Fig. 3g and Additional file 1: Fig. S21)**.** Furthermore, we compared the EV COSMIC mRNA transcript levels to TCGA pancreatic cancer data (Fig. 3h) to assess the degree of similarity between EV and TCGA transcripts. We showed ExCy obtains much more COSMIC-associated mRNA transcripts than the other methods and was more representative on the TCGA profile (indicated in pink).

### Landscaping functional pathways and differential expression analysis to reveal pancreatic tumor derived EVs

**Fig. 4:**
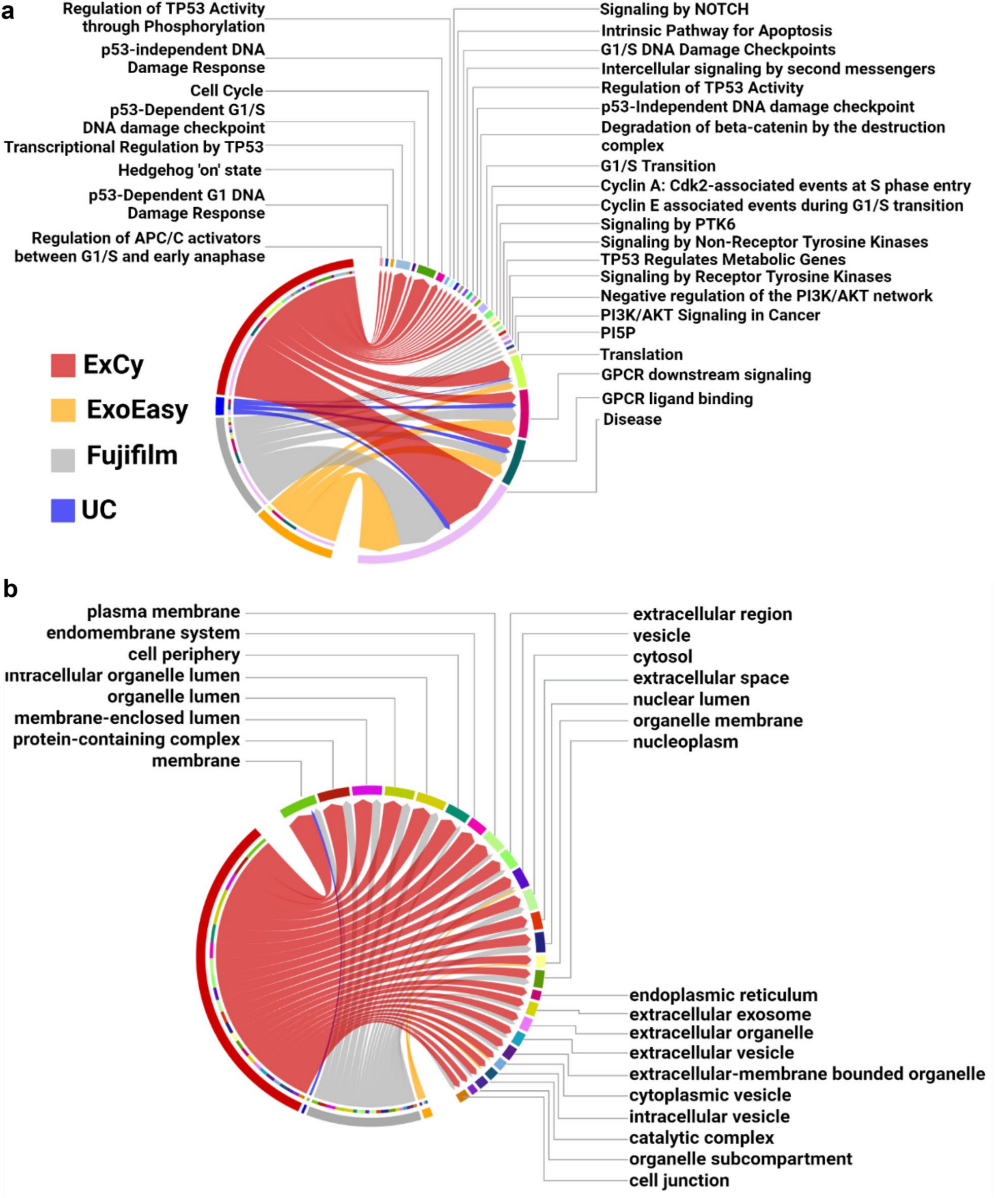
Differential analysis and statistical comparisons on method-dependent mRNA transcript levels to reveal the pancreatic tumor derived EVs specifically isolated from ExCy method. **a** Chord plot illustrating the pancreatic cancer related pathways associated with method dependent EV-mRNA transcripts. The edge size in the chord plot represents the number of annotated mRNAs found in each pathway. **b** Chord plot illustrating the top 25 statistically significant cellular component pathways found by gProfiler after pooled differential analysis from all the EV isolation methods by edgeR on pancreatic cancer patients (n = 4) and healthy control (n = 3), then ranking differential transcripts to each isolation method across all patient samples (n = 7)

We investigated the disparity level of EV-mRNA transcripts and their dependency on isolation methods for biomarker discovery in pancreatic cancer. We performed pathway analysis on the unique mRNA annotations in each patient sample across all isolation methods, relative to cancer and pancreatic cancer pathways (Additional file 5), then tabulated the unique and shared pathways per method across all patients (Fig. 4a). Each method contains distinct cancer-related pathways, but the shared pathways (i.e. Translation, GPCR ligand binding, GPCR downstream signaling, and Disease) for the methods indicated different mRNA transcripts contributing to the pathway’s regulation. We further analyzed the pancreatic cancer-associated, method-specific mRNA transcripts in the shared pathways by using TCGA’s Cancer Gene Consensus analysis on each pathway to determine pancreatic cancer significance. The only overlapping pathways that were assessed by TCGA’s Cancer Gene Consensus analysis to have pancreatic cancer association from EV mRNAs was “Disease”. The ExCy and Fujifilm’s mRNA transcripts in the Disease pathway were revealed by TCGA’s Cancer Gene Consensus analysis to be more likely mutated: *MUC4, CDK4,* and *CD79A, ARAF*, respectively. To further validate whether those mutated genes come from non-EV contamination, we tabulated all differential transcripts in the healthy and tumor sample sets, then ranked the differential mRNA transcripts to an isolation method, and applied pathway analysis specifically for gene ontology, cellular component (GO:CC), because GO:CC is the pathway specific for extracellular vesicle ontology as shown in Fig. 4b (Additional file 3). These results further support that ExCy isolates transcripts mostly relevant to EV-specific terms and exosome origin, indicating a higher isolation homogeneity compared to other methods. In contrast, UC is only enriched for the membrane GO term (GO: 0016020). Given how EV membrane profiles resemble their parent cells, the EV transcriptomic profile could reveal their cellular origin. Since our differential analysis evaluates method-dependent mRNA transcript levels through statistical comparisons, the results from ExCy-isolated EVs offered the highest relevance to pancreatic tumors and originating cellular components, indicating the highest EV isolation quality.

**Fig. 5:**
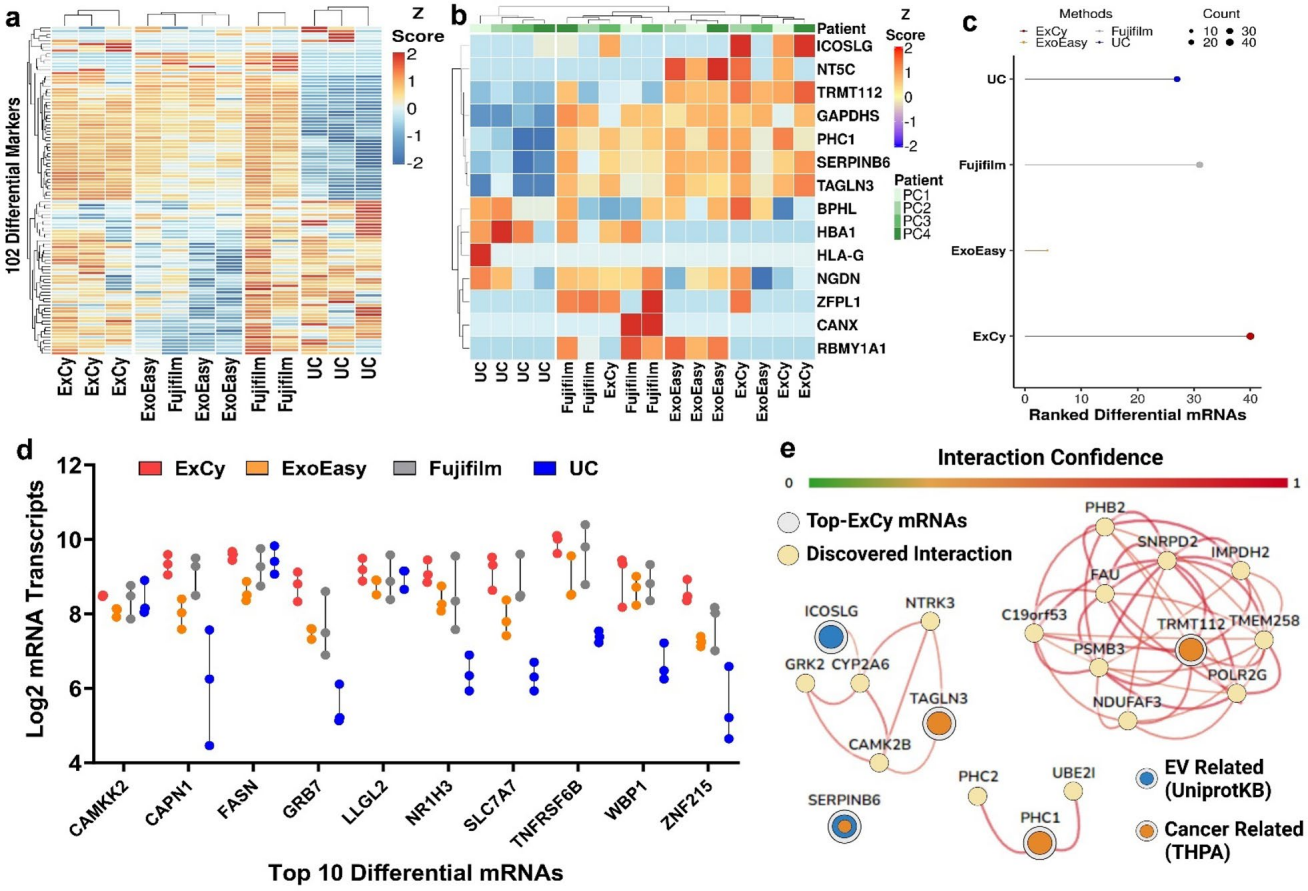
Differential expression analysis of mRNA profiles derived from different EV isolation methods. **a** Heatmap showing the landscape of differentially expressed transcripts in healthy patient plasma isolated EVs across different isolation methods, as determined by edgeR (FDR < 0.05). The color gradient indicates the Z-score. Hierarchical clustering was performed using Ward’s D2 method [75]. **b** Heatmap showing the landscape of differentially expressed genes in pancreatic cancer patient plasma isolated EVs across different isolation methods, as determined by edgeR (FDR < 0.05). **c** Differential transcripts in healthy patients were ranked across each method based on their average transcript count. The filled point represents the total number of differentially ranked mRNAs in the isolation method, while the line segment helps visualize the relative difference of total differentially ranked mRNAs between methods. **d** ExCy’s top 10 ranked differential mRNA transcripts compared to other EV isolation methods illustrated by the box-and-whisker plot. **e** ExCy ranked mRNA transcripts were inputted into HumanBase’s GIANT global tissue network. Input mRNA transcripts were further identified as EV related by UniprotKB or cancer related by The Human Protein Atlas, while the connections between nodes represent the interaction confidence greater than 0.60

We analyzed differential expressions using edgeR to investigate the statistically significant differences in the method-dependent mRNA transcripts between the healthy group (Fig. 5a) and the pancreatic cancer patients (Fig. 5b). In healthy patients, all methods appear to obtain statistically similar mRNA transcript levels, except for 102 mRNAs with differential expressions from the hierarchical clustering analysis shown in Fig. 5a. Notably, the differential expression pattern from the UC group is statistically different from ExCy, Fujifilm, and ExoEasy groups, while Fujifilm and ExoEasy did not show statistical difference from each other (Additional file 1: Table S5). In consideration of each isolation method’s procedure, our observation also suggests the potential contamination by non-EV structures associated with UC and Fujifilm’s EV isolation. This observation is also consistent with our EQI determination in Fig. 2f and Additional file 1: Fig. S11 and S12 from the ExoView analysis. Based on our findings, we ranked each method's differential mRNA transcript levels within the healthy sample set (Fig. 5c) and then plotted the top 10 ExCy-ranked mRNAs (Fig. 5d) to assess the trending differences in mRNA profiles between the EV isolation methods. We observed that ExCy consistently identified a significant EV marker, CAPN1. When queried by UniProtKB, CAPN1 corresponds to the Extracellular Exosome pathway. Next, we investigated the tumor cohort (Fig. 5e) and observed a smaller number of differentially expressed genes between each EV isolation method. As observed consistently with the healthy sample cohort, the UC derived transcriptomic profile from tumor group significantly differs from ExCy, ExoEasy, and Fujifilm groups (Fig. 5b). We extracted all ExCy ranked differential tumor transcripts and performed functional enrichment analysis using the HumanBase enrichment tool and global functional interaction network (Fig. 5e). HumanBase’s global functional interaction network identified ICOSLG to show an interaction relationship with CYP2A6, which is a critical network of drug metabolism for monitoring drug responses. However, this interaction may not be detected using ExoEasy, Fujifilm, or UC isolated EVs to quantify mRNA transcript levels. Interestingly, TAGLN3, a potential oncogene and regulator of RNA Polymerase II, was detected by ExCy, ExoEasy and Fujifilm, but not by UC. Similarly, for TRMT112, neither Fujifilm nor UC may detect this transcript, missing TMRT112’s impact as an oncogene and subsequent role as a macromolecular methylator and rRNA effector. Finally, PHC1 and SERPINB6 were not detected by UC; however, these two genes play essential roles as either a pancreatic-cancer specific oncogene, regulating GMNN expression in the pancreas, or potential EV-associated oncogene, as a serine protease, respectively (Additional file 1: Fig. S21). The ExCy isolation method showed the highest average and least variable GMNN transcript abundance, suggesting further evidence for higher EV homogeneity. Our findings also highlight the necessity of using an additional optimal EV isolation method to validate identified markers for functional analysis.

### High precision identification of early-stage pancreatic tumor markers from circulating EVs

**Fig. 6:**
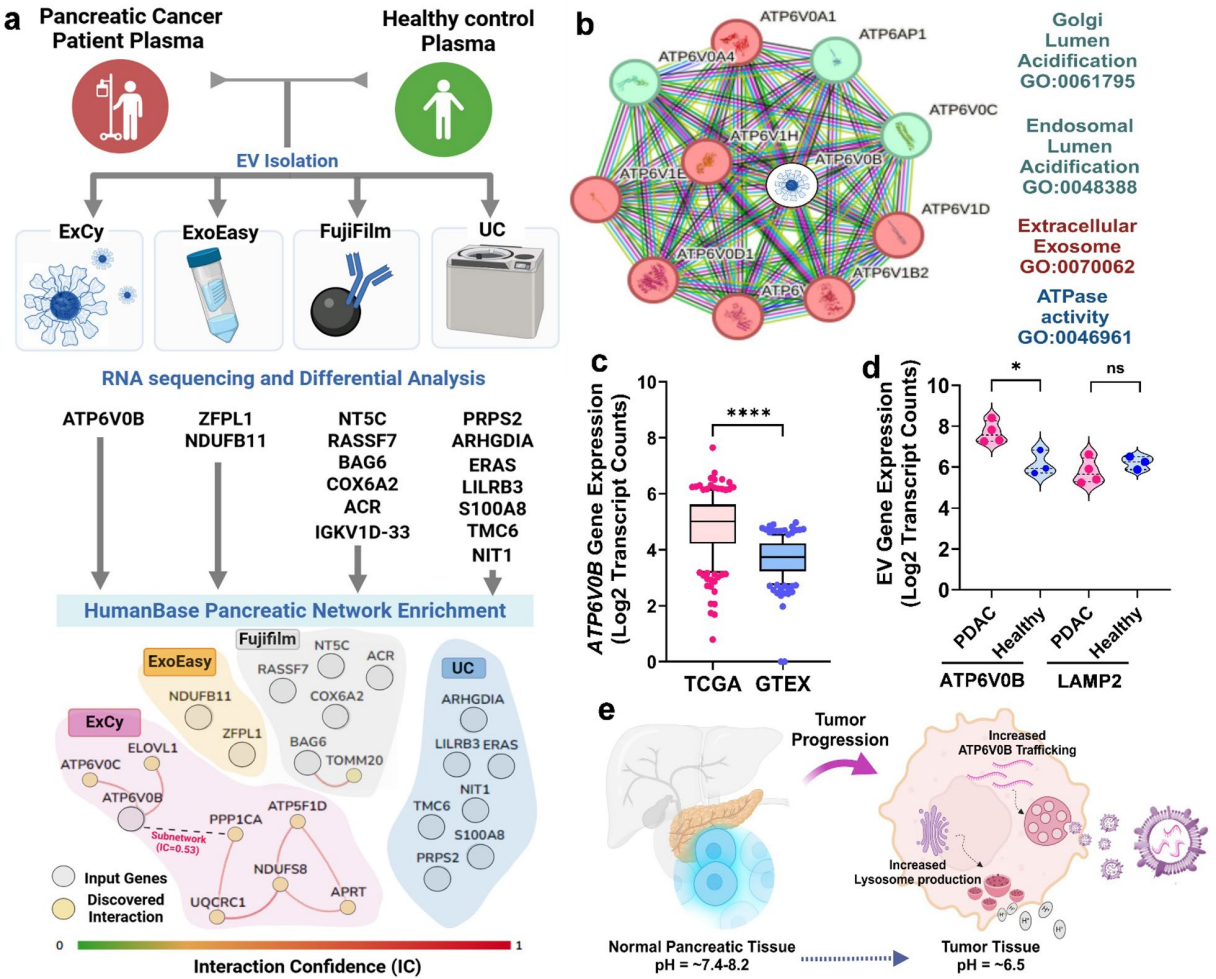
Differential expression analysis indicates ExCy’s capability to identify specific pancreatic tumor associated marker. **a** Differential analysis was applied to compare tumor versus healthy samples to find distinct markers (edgeR FDR < 0.1) per method. We then pooled all the differential markers into the HumanBase GIANT pancreatic network for enrichment analysis to discover interactions within the pancreas. The connections between nodes represent the interaction confidence greater than 0.60. Only ExCy (*ATP6V0B*) and Fujifilm (BAG6) differential transcripts were deemed valid based on their connection within the network. **b** We queried STRING on ATP6V0B from ExCy EV isolation to investigate protein interactions and their representative ontological terms as a tumor associated marker. K-means was performed to understand subnetwork protein–protein interactions, where green and red colored nodes indicate the cluster assignment by K-means. **c** Population analysis of ATP6V0B gene expression by comparing bulk-RNA signatures from non-disease genome-tissue expression database (GTEX) and Cancer Genome Atlas database (TCGA) with PDAC samples. Differential analysis was performed by edgeR (FDR < 0.05). Batch correction was performed through ComBat-seq [79]. **d** Expression analysis for ExCy derived EV-ATP6V0B and LAMP2 gene expression. **e** Schematic illustration of proposed mechanism for ATP6V0B gene expression in pancreatic tumor derived EVs. The tumor-associated EVs carry a higher *ATPV0B* signal to communicate acidosis compared to healthy cells

We hypothesized that markers identified by an EV isolation method must be associated with EV biology within the context of pancreatic cancer, which we considered testable through functional enrichment analysis after differential analysis. Given the limited sample number to discover tumor associated markers in each method, we slightly adjusted the FDR from 0.05 to 0.10 to enable a wider pool of markers to be analyzed. Despite the implicit technical bias associated with each isolation method, we expected to find overlapping differential markers since we applied these methods to the same samples. Strikingly, none of the observed differential markers across the methods overlapped (Fig. 6a). Under the assumption that all methods obtain EV transcripts, we pooled all the differential markers together for pancreatic enrichment analysis by HumanBase to ascertain functional potential. At an interaction confidence threshold of 0.60, HumanBase revealed that ExoEasy and UC’s identified markers did not interact with the pancreatic network. However, both ExCy derived *ATP6V0B* and Fujifilm derived *BAG6* markers showed strong interconnection in pancreatic tissue. Interestingly, a subnetwork within HumanBase remained present above the threshold from ExCy prepared EVs, suggesting much larger regulatory impact from *ATP6V0B* than the Fujifilm-identified marker, *BAG6*. We further investigated ExCy’s and Fujifilm’s identified markers by looking at STRING’s functional protein network for *ATP6V0B* (Fig. 6b) and *BAG6* (Additional file 1: Fig. S22), respectively. In STRING, we used an interaction confidence threshold of 0.90 to ensure maximum confidence regarding the marker’s biological capacity. We extracted their gene ontology (Additional file 6), revealing that ExCy discovered marker, *ATP6V0B*, is a highly relevant EV marker that increases acidification (logFC = 2.02) of the endosomal and Golgi lumen in pancreatic tumors, dysregulating both EV production and cytosolic ion-channel gradients. On the other hand, Fujifilm discovered marker, *BAG6*, is a cytosolic molecular chaperone participating in delivering proteins to the endoplasmic reticulum or proteasome, unspecific to the EV biology.

We further applied a population comparison of *ATP6V0B* gene expression using pancreatic tissue from UCSC’s Xena database to compare non-diseased Genome-Tissue Expression (GTEX) [76] and TCGA’s PDAC (Additional file 1: Fig. S23), which confirmatively showed a significant increase of specific *ATP6V0B* exon expression in the cancer group (Fig. 6c), consistent with the EVs isolated by ExCy (Fig. 6d, and Additional file 1: Fig. S6 and S23). *ATP6V0B*, as the acidification functional gene, is particularly impactful in pancreas tissue which requires an alkaline milieu to function properly; therefore, low pH promotes their dysfunction, potentially the tumorigenesis (Fig. 6e) [77]. We reasoned that pancreatic tumor cell secreted circulating EVs may transport ATP6V0B and undergo acidosis by increased V-ATPase subunits [50, 78]. Although LAMP2 is also a well-recognized EV marker associated with cancer, we observed slight significance for differentiating between PDAC and healthy group (Fig. 6d). Therefore, these observations strongly supported that ATP6V0B identified by our ExCy isolation and statistical algorithm pipeline is specifically associated with EV biology within the context of pancreatic cancer, indicating a successful discovery of novel early-stage pancreatic tumor marker.

**Fig. 7:**
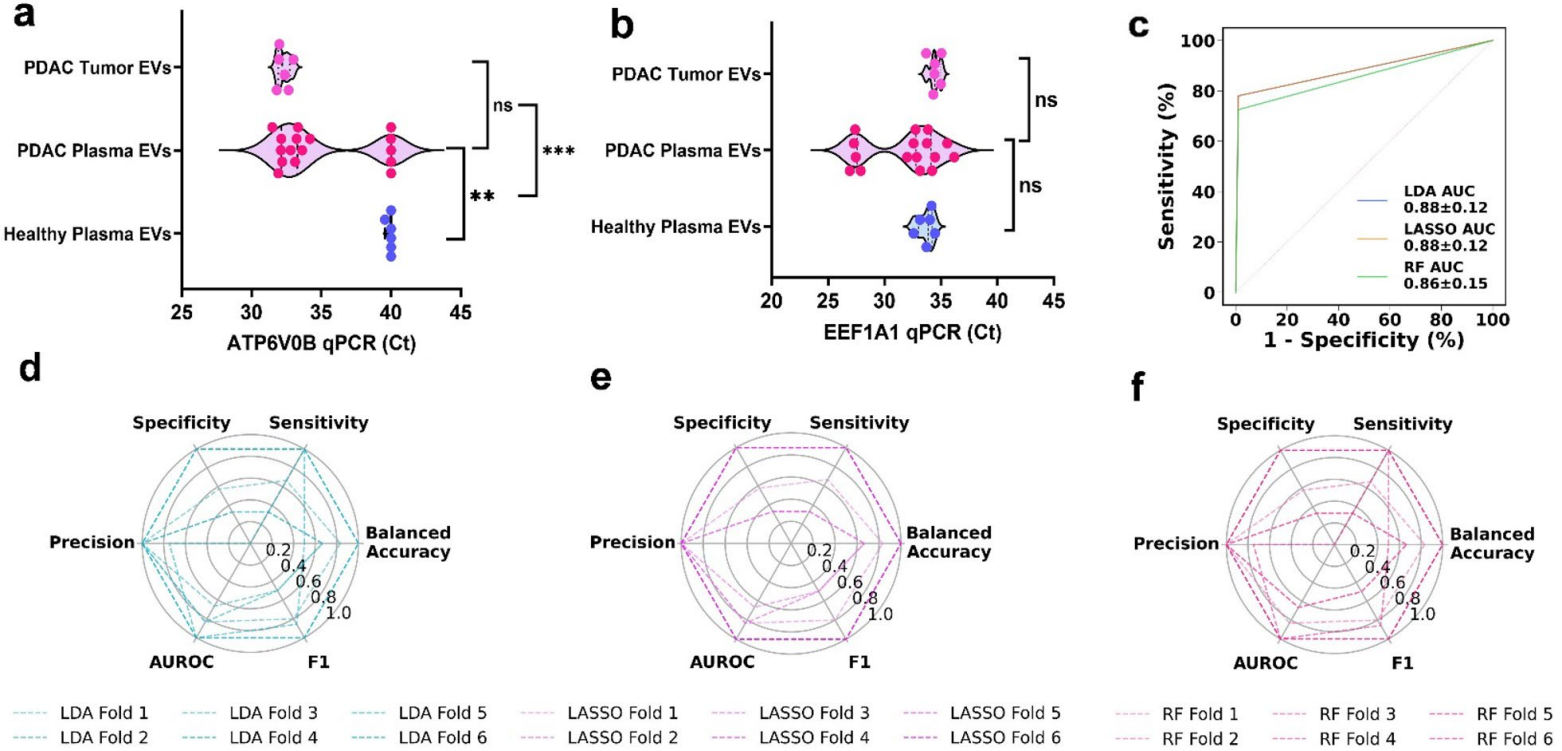
**a** qPCR detection of ATPV06B from ExCy isolated EVs in early-stage PDAC patient plasma and healthy controls, along with matched pancreatic tumor tissue, compared with positive control marker EEF1A1 in (**b**). **c** The receiver operating curve (ROC) analysis to ascertain EV derived *ATP6V0B* may serve as a diagnostic biomarker for early-stage pancreatic cancer patient liquid biopsy with LDA, LASSO, and random forest (RF) model performance shown as a radar plot in (**d**), (**e**), and (**f**), including the sensitivity, specificity, balanced accuracy, F1, AUROC, and precision

To validate *ATP6V0B* as a pancreatic tumor marker from circulating EVs for liquid biopsy, we collected plasma samples (N = 22) from 16 early-stage pancreatic cancer individuals, of which 6 had matched pancreas tumor tissues, and 6 healthy samples (Additional file 1: Table S6 for patient information) for quantitative polymerase chain reaction (qPCR) detection. Using our differential exon analysis from the FREYA pipeline to locate the region of interest for determining the ATP6V0b target (Additional file 1: Fig. S24). Furthermore, we validate the PCR results on ATP6V0B gene from isolated plasma EVs using ExCy compared with the detection from subsequent EV-depleted plasma (Additional file 1: Fig. S25), which confirms our resultant ATP6V0B measurement is from plasma EVs. Next, we performed our qPCR by normalizing all cDNA concentrations to the health cohort in detecting ATP6V0B (Fig. 7a) and the internal control marker, EEF1A1 (Fig. 7b). In contrast to the internal control gene EEF1A1, we observed the cycling threshold (Ct) for detecting ATP6V0B was statistically significant for EVs isolated from pancreatic cancer plasma and tumor tissue, when compared to the healthy cohort. Although the internal control was not statistically different across the groups, the internal control’s coefficient of variation shown in the PDAC plasma EVs is higher than expected due to both patient bias and EV heterogeneity, despite equivalent stoichiometric sample input into qPCR. Thus, we used Ct value, instead of 2^ΔCt, which may be more appropriate for EVs. The level of ATP6V0B gene is comparable between pancreatic tumor derived EVs and PDAC patient plasma derived EVs. We also leveraged LDA, LASSO, and random forest to evaluate *ATP6V0B*’s potential as a biomarker for early-stage pancreatic cancer diagnosis (Fig. 7c). Through using a sixfold stratified cross-validation scheme, we showed all models obtain a mean AUROC value of 0.86–0.88, while additionally high performances through the folds (Fig. 7d–f), including precision, specificity, selectivity, balanced accuracy, and F1. Evaluating the clinical significance, we showed that the random forest model classified metastatic vs non-metastatic cases with an AUROC average of 0.83 (Additional file 1: Fig. S27). Furthermore, we identified that ATP6V0b’s Ct in plasma EVs has moderate correlation to a patient’s tumor stage (Pearson r = − 0.44; Additional file 1: Fig. S28). These results indicate that ATP6V0B may be a specific clinical marker for detecting early-stage pancreatic cancer through circulating EVs, which opens a new avenue for liquid biopsy diagnosis of pancreatic cancer.

## Conclusion

EVs are increasingly used to understanding regulatory mechanisms and their use in clinical research, which has resulted in substantial clinical trial establishment [3]. However, each component of the EV pipeline contributes to the observed EV heterogeneity [3, 7, 20, 21, 23, 25, 26, 80] and this heterogeneity has far-reaching implications, most notably in terms of the interpretations drawn from EV samples concerning biological processes and their reproducibility [7, 8, 21, 25, 29–35]. EV isolation induced heterogeneity may lead to conflicting conclusions and hinder the ability to replicate findings, thereby impeding the overall progress of clinical research. We first addressed EV heterogeneity at the isolation level by developing a novel strategy to isolate EVs using an immunomagnetic capture-release approach, which can enhance the selectivity for EV isolation, due to the well-observed selectivity to insert into unilamellar particles [39–41, 64–67], in addition to ExCy’s unique NanoPom topography [36] capture with nano-scale cavities. The capture-release process also could reduce contamination by lipoproteins and nano-debris, as observed by the ExoView (Additional file 1: Fig. S8–S11) and Simple Western assay (Additional file 1: Fig. S12).

We created a generalizable EV quality metric for comparison between EV isolation methods by employing data science techniques to define the extent of EV heterogeneity, which we demonstrated in human plasma samples. Using this framework, we established a statistical benchmark for the expected EV isolate quality, demonstrating how statistical standardization was essential to directly evaluate EV quality across isolation methods. Our statistical benchmark supports that ExCy enables broad applications for isolating EVs from various biological fluids, which contributes to high-quality EV isolation. Moreover, we further suggest an optimal EV isolation method that is crucial in biomarker discovery to validate identified markers for functional analysis, to which direct comparison between isolation methods is absolutely required, as we demonstrated our generalizable metric by comparing 4 different total EV isolation methods (ExCy, ExoEasy, Fujifilm, and UC). We specifically chose total EV isolation methods aiming to obtain high-quality homogenous EV isolates, despite mechanistic differences in isolation strategy. ExCy captures EVs by transmembrane insertion, ExoEasy isolates by membrane affinity and centrifugation, Fujifilm by phosphatidylserine affinity, and UC by density, each in distinct EV isolate profiles despite the same EV source. Though MISEV provides semi-quantitative metrics to investigate EV quality, even when considering the same source, these metrics were confounded by the isolation method. Therefore, we adjusted for method-specific technical bias using quantile–quantile normalization to adjust their resulting measurements. Quantile transformation rescales measurements between 0 and 1 based on the cumulative distribution function, defining all values equally likely. Allowing an equal likelihood of measurement, when given the same EV source, enables statistical generalization across different EV isolation methods (Fig. 2). When we applied the quantile transformation, we did not observe any loss of information. Instead, we demonstrated that our approach coincided with the field's understanding that EV isolates are still method dependent and signify inconsistent observations. Further, we demonstrated that comprehensive characterization is required to indicate an EV isolate’s quality before subsequent translational analysis and that relying on paired measurements has likely obfuscated potential discoveries for confounding existing conclusions reported in clinical research. To address this issue, we laid a foundation for characterizing an EV isolation quality by demonstrating that the EQI could reveal a method’s expected EV isolate homogeneity upon resampling. If we included the studies from EV-TRACK, our statistical metric may enable EV quality comparisons across EV isolation methods and substantially improve the overall rigor in clinical research.

We validated ExCy’s high-quality EV isolation with next generation sequencing, showing the differences in RNA transcript quality through the FREYA pipeline with HISAT2 (Additional file 1: Table S2) and FastQC (Additional file 3). EV transcriptomics are subject to confoundment regarding both EV heterogeneity and transcriptomic practices. Using FastQC, we showed a significant difference in library quality after processing. We observed differences in the total RNA and mRNA distribution between the EV isolation methods; however, the correlated mRNA transcript levels between all methods were moderate (Pearson’s r = 0.78, Additional file 1: Fig. S18). On the other hand, we showed through differential analysis that all methods are statistically similar in the bulk of their EV isolate mRNA transcript levels, differing by a distinct fraction of mRNA transcripts, though this observation may be affected by the limited sample power in our study. Based on the mRNA transcript levels, pathway analysis, and differential analysis, we showed that ExCy may be the most useful for developing clinical applications.

We also proposed that EV isolation method-specific markers in disease contexts must need to be associated with EV biology, which we demonstrated through functional enrichment of the differential analysis. Total EV isolation methods are expected to pull down similar EV transcripts, given that these methods are EV selective; however, we showed that regardless of isolation technique, the expected EV quality strongly affects differential analysis to find relevant markers (Fig. 6a), since no overlap in differential markers were identified. To draw a confirmative conclusion, we cross-validated HumanBase’s enrichment analysis with STRING’s functional protein networks (Fig. 6b), and population analysis with GTEX and TCGA (Fig. 6c) compared to EVs (Fig. 6d), showing that ExCy isolated EV marker *ATPV06B* was highly significant for EV biology in the pancreatic cancer disease context (Fig. 6e). We also assessed the diagnostic value of ATPV06B in circulating EVs using a pilot cohort of 22 plasma samples, 16 from pancreatic cancer and 6 from healthy individuals. PDAC is one of the most lethal cancers, with a 5-year survival of < 13%, which is often owning to the diffuse symptoms resulting in late diagnosis. Serum CA19-9 has been the most evaluated biomarker for PDAC, however, it suffers from inadequate specificity with elevated levels in several other indications. Our approach yields improved sensitivity and specificity using *EV ATP6V0B* as the biomarker detected from stage I and stage II PDAC patient plasma. However, further investigation is still necessary to fully validate *ATP6V0B* as a pancreatic cancer biomarker for large-scale cohort.

In summary, we addressed a significant challenge in clinical translation of EV research by developing an algorithm integrated isolation pipeline for high precision biomarker discovery. The rigorous and generalizable statistical metric to evaluate EV isolate quality ensured the transcriptomics for deriving a novel early-stage PDACA diagnostic marker. For biomarker discovery by transcriptomics, we suggest increasing both technical and biological replicates, and include an EV isolation control to support translational claims. Importantly, while we demonstrate all methods isolating EVs in some capacity, EV isolation methods should be controlled with another isolation technique to avoid conflicting outcomes in clinical research, and UC may be unable to serve as the reference or benchmark method, revealing itself as an outlier in this study. In this outlook, ExCy’s utilization in broad EV basic and clinical research as an isolation method could provide the least confounding evidence to support biological conclusions and advancement towards clinically translatable outcomes.

## Supplementary Information

**Additional file 1**. Supplementary Figs. 1–28 and Tables 1–6.

**Additional file 2**. ExoQuality Index Dataset and Sequencing information. The dataset used to compute the EQI and sequencing information regarding HISAT2’s RNA annotations per sample across all methods before and after Vesiclepedia mapping. FastQC reports – External quality reports using FastQC, before and after applying trimmomatic, detailing each EV isolation’s transcriptomic sample assembly right after sequencing by the NovaSeq 6000. Each report will present basic assembly statistics followed by listing quality metrics including, Per base sequence quality, Per tile sequence quality, Per sequence quality, Per base sequence content, Per sequence GC content, Per base N content, Sequence Length Distribution, Sequence Duplication levels, Overrepresented sequences, and adaptor content.

**Additional file 3**. gProfiler’s enrichment analysis. Enrichment analysis was applied to each EV isolation’s transcriptomic sample based on their unique mRNA annotations.

**Additional file 4**. EV Isolation method-specific mRNA annotations and subsequent Reactome pathway.

**Additional file 5**. Patient STRING pathways and intersecting mRNA annotations.

**Additional file 6**. STRING pathway analysis on BAG6 and ATPV06B.

## Data Availability

All raw and processed sequencing data generated by this study is available at the NCBI Gene Expression Omnibus (GEO; https://www.ncbi.nlm.nih.gov/geo/query/acc.cgi?acc=GSE246925). Computer code used in this manuscript is available at github.com/zfg2013/ExCy. All code used in this manuscript is shared at this repository, and to ensure reproducibility, we provide a README with version information for each tool plus any parameter settings used in the data processing and analysis.
