# Supplementary material for: Nanomaterial isolated extracellular vesicles enable high precision identification of tumor biomarkers for pancreatic cancer liquid biopsy
Source: J Nanobiotechnology. 2025 Jul 1;23:467. doi: 10.1186/s12951-025-03527-3 (PMC12211367; doi:10.1186/s12951-025-03527-3)
Supplement: Supplementary file 1 — Additional file 1. Supplementary Figs. 1–28 and Tables 1–6. [file 12951_2025_3527_MOESM1_ESM.docx]

Supplementary Materials

**Nanomaterial Isolated Extracellular Vesicles Enable High Precision Identification of Tumor Biomarkers for Pancreatic Cancer Liquid Biopsy**

Zachary F. Greenberg^1^, Samantha Ali^1^, Andrew Brock^1^, Jinmai Jiang^1^, Thomas D. Schmittgen^1^, Song Han^2^, Steven J. Hughes^2^, Kiley S. Graim^3^**^*^**, Mei He^1^**^*^**

1. Department of Pharmaceutics, College of Pharmacy, University of Florida, Gainesville, Florida 32610, United States
2. Department of Surgery, College of Medicine, University of Florida, Gainesville, Florida 32610, United States
3. Department of Computer & Information Science & Engineering, Herbert Wertheim College of Engineering, University of Florida, Gainesville, Florida, 32610, USA

***** corresponding contact

**This PDF file includes:**

Supplementary Figs. 1-28 and Tables 1-6.

**Other Supplementary Materials for this manuscript include the following:**

Supplementary File 1 – ExoQuality Index Dataset and Sequencing information. The dataset used to compute the EQI and sequencing information regarding HISAT2’s RNA annotations per sample across all methods before and after Vesiclepedia mapping.

Supplementary File 2 – FastQC reports – External quality reports using FastQC, before and after applying trimmomatic, detailing each EV isolation’s transcriptomic sample assembly right after sequencing by the NovaSeq 6000. Each report will present basic assembly statistics followed by listing quality metrics including, Per base sequence quality, Per tile sequence quality, Per sequence quality, Per base sequence content, Per sequence GC content, Per base N content, Sequence Length Distribution, Sequence Duplication levels, Overrepresented sequences, and adaptor content.

Supplementary File 3 – gProfiler’s enrichment analysis. Enrichment analysis was applied to each EV isolation’s transcriptomic sample based on their unique mRNA annotations.

Supplementary File 4 – EV Isolation method-specific mRNA annotations and subsequent Reactome pathway.

Supplementary File 5 – Patient STRING pathways and intersecting mRNA annotations.

Supplementary File 6 – STRING pathway analysis on BAG6 and ATPV06B


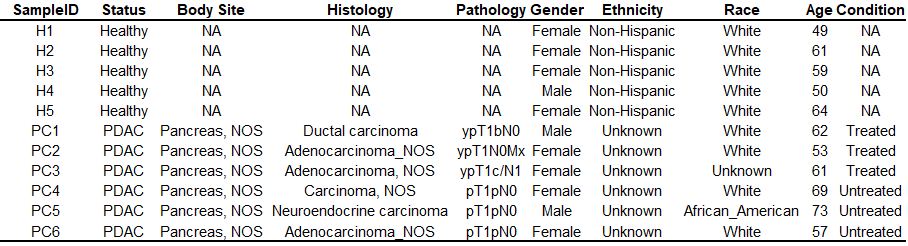
**Table s1**. The discovery cohort used to isolate EVs across the methods and determine ATP6V0b.


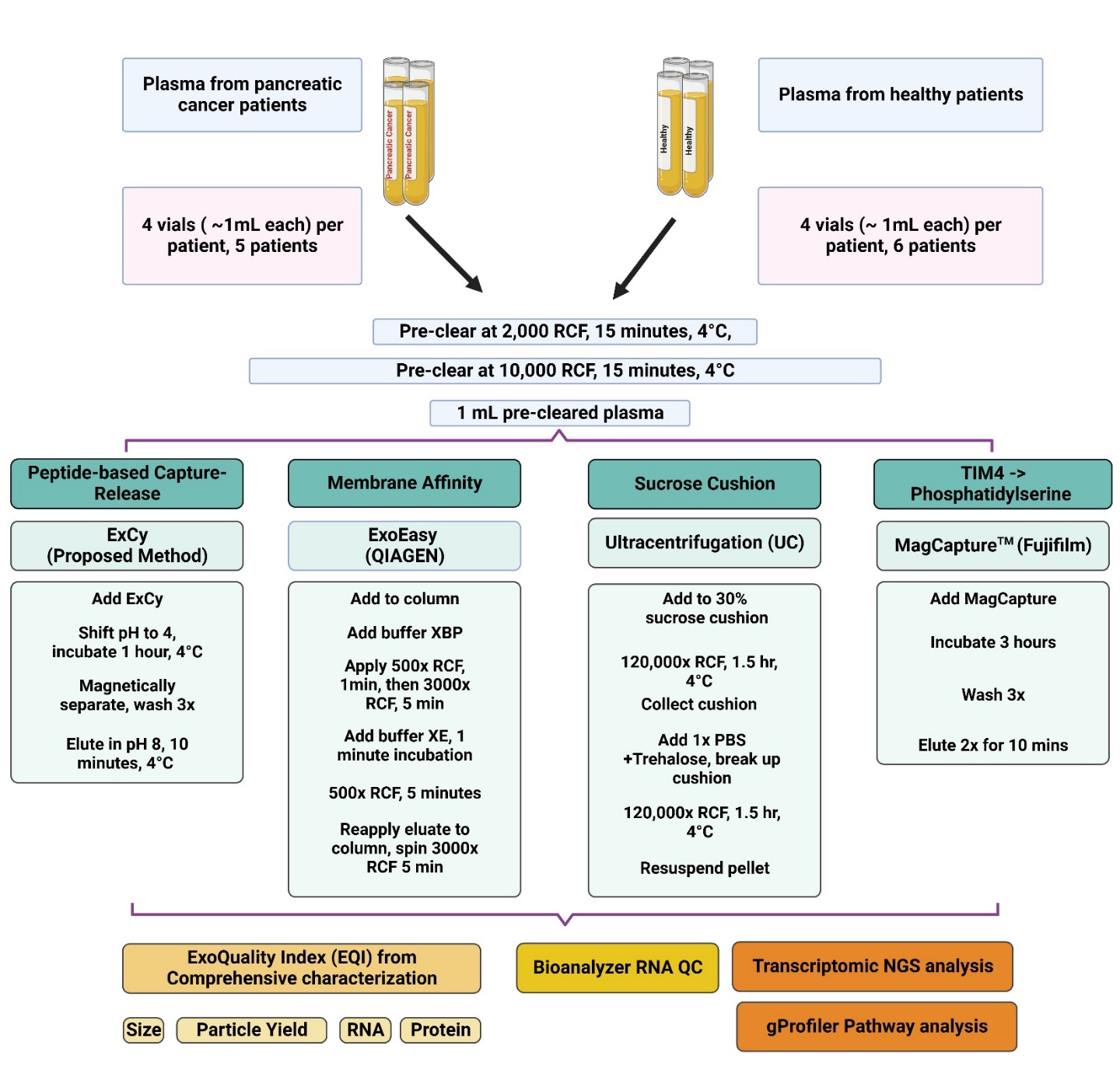


**Figure s1.** Workflow of sample preparation of EVs from human patient plasma associated with the quality index assessment and transcriptomic NGS study.


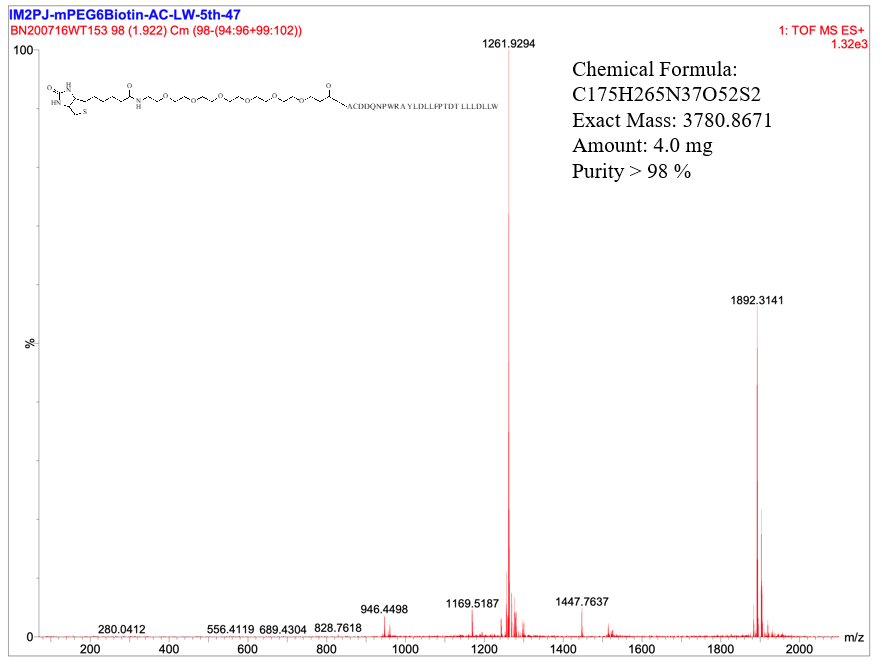


**Figure s2**. The mass spectrometric characterization and QC after peptide microwave synthesis.


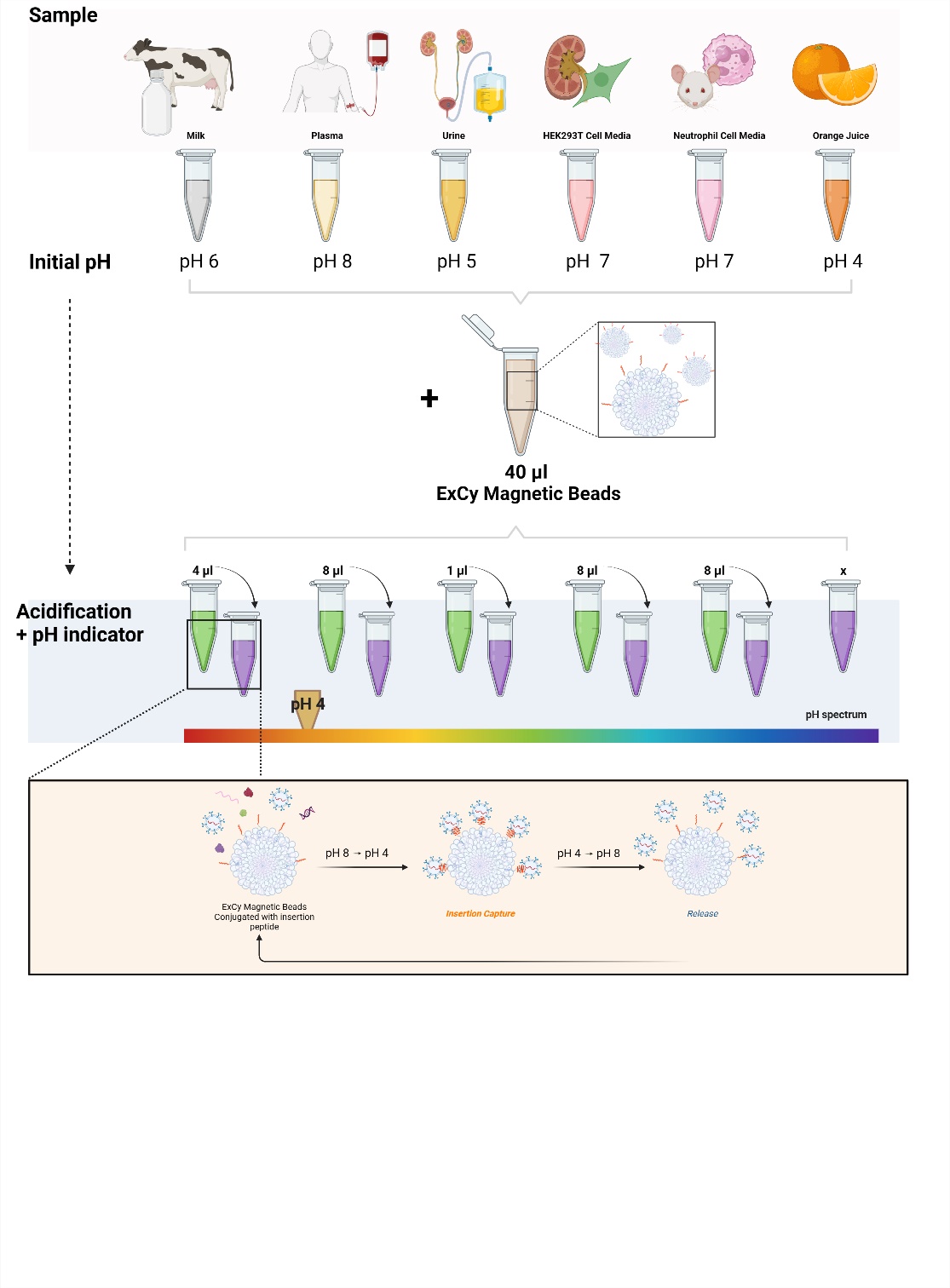


**a**

**c**

**b**


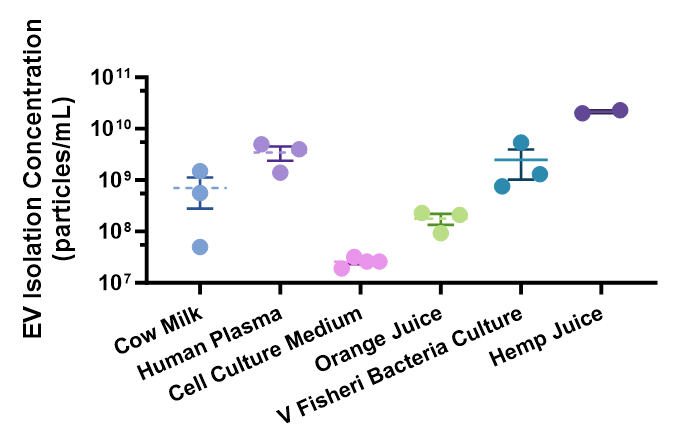


**Figure s3.** **a)** The workflow of broad applicability of ExCy on isolating EVs from various biological media. **b)** ExCy isolation of EVs from a variety of biological fluids to prove the reproducibility and applicability of developed isolation approach. n=3-4 and CV=~3-5%. **c)** Zeta View nanoparticle tracking analysis on size of extracted EVs from various fluids.

**a**


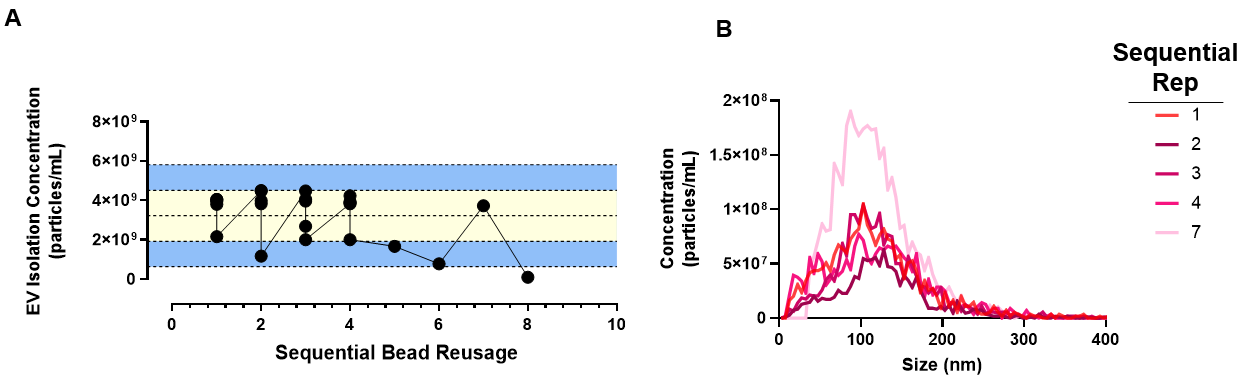


**b**


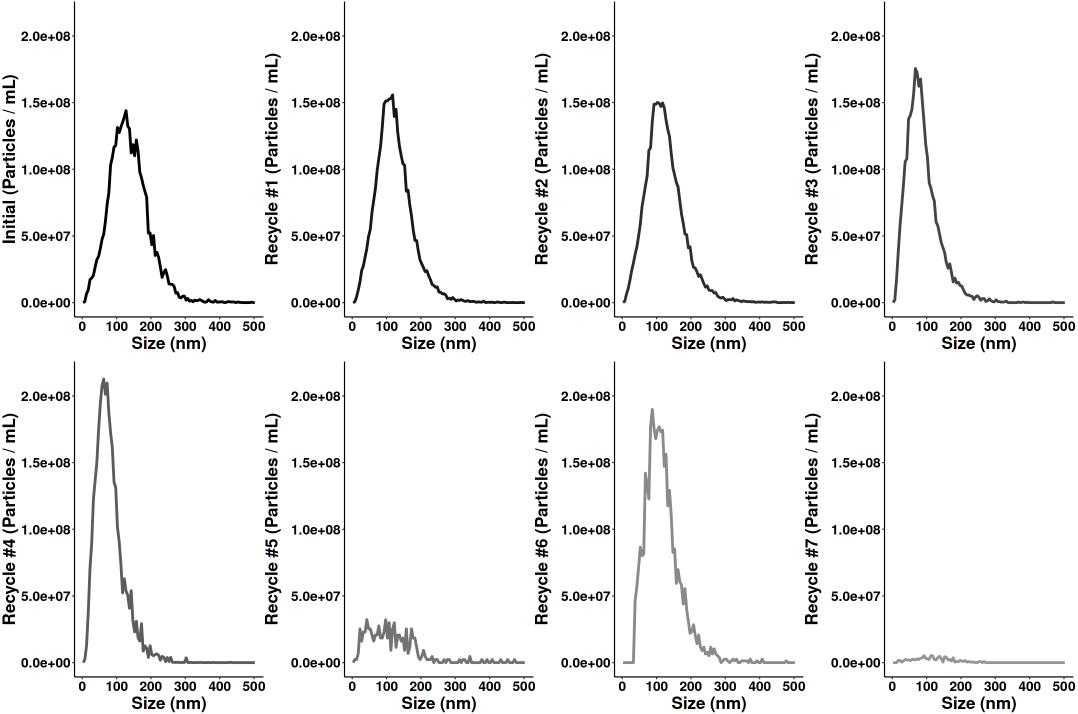


**Figure s4**. ExCy’s capability to recapture EVs. **a)** EV isolate concentrations after ExCy’s sequential usage in the same sample type (plasma), indicating the isolation reproducibility. Yellow bars indicate 95% confidence interval, while blue bars fall out of range. **b)** Nanoparticle tracking analysis for each sequential isolation. Initial designates the starting EV isolate concentration from human plasma.

**
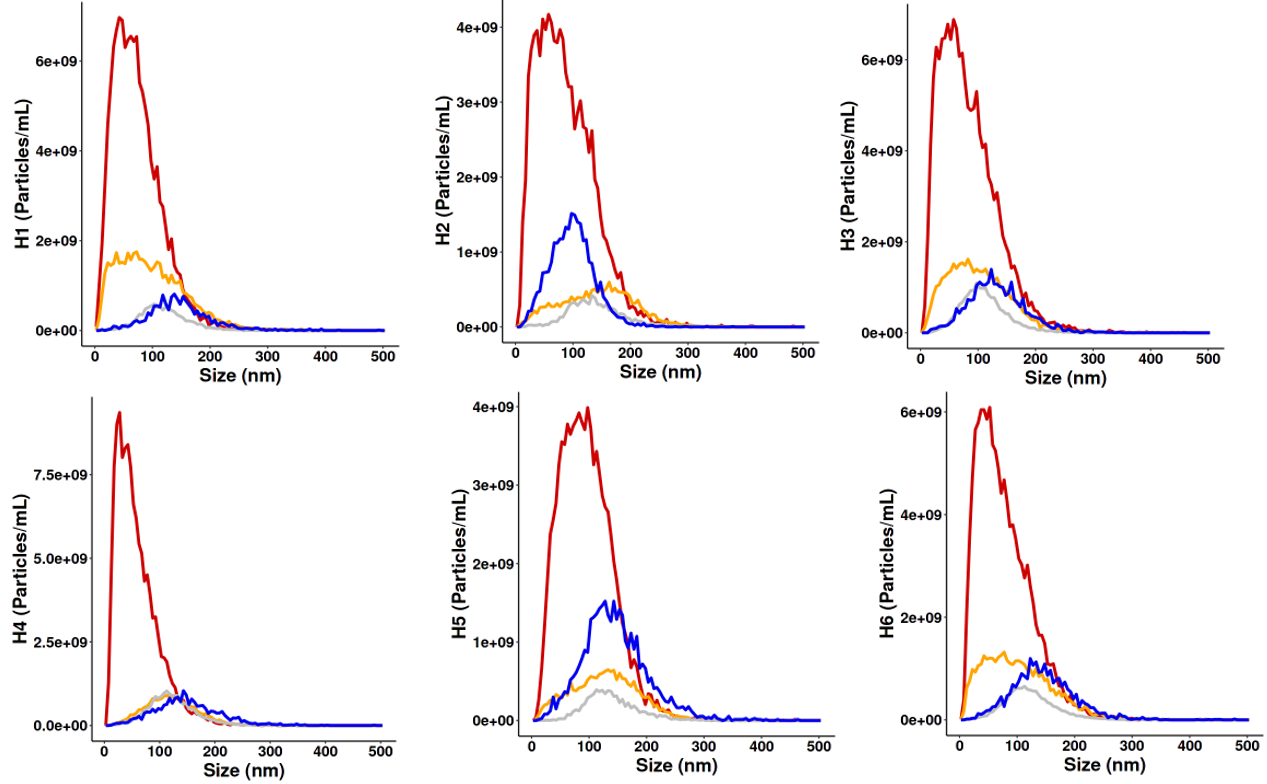

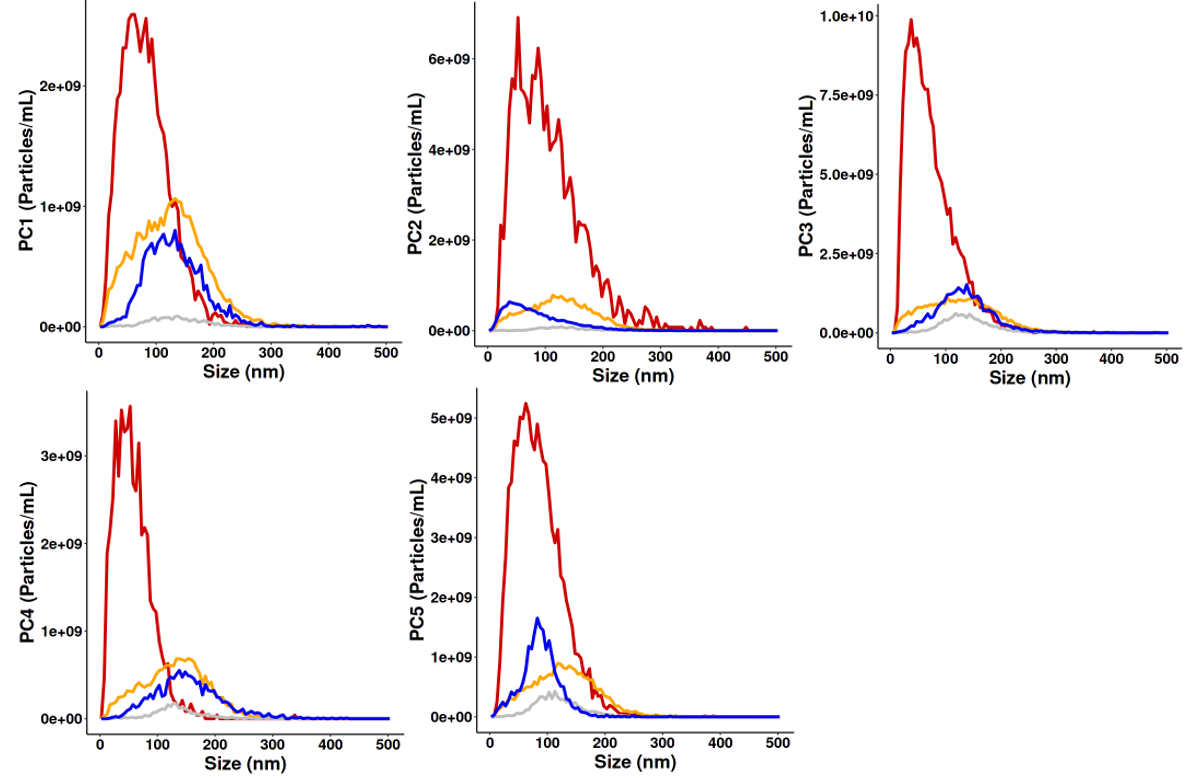
Figure s5**. NTA size distribution profile from all human plasma samples from **top:** 5 Pancreatic cancer patient plasma samples, and **bottom:** 5 Healthy control plasma samples.


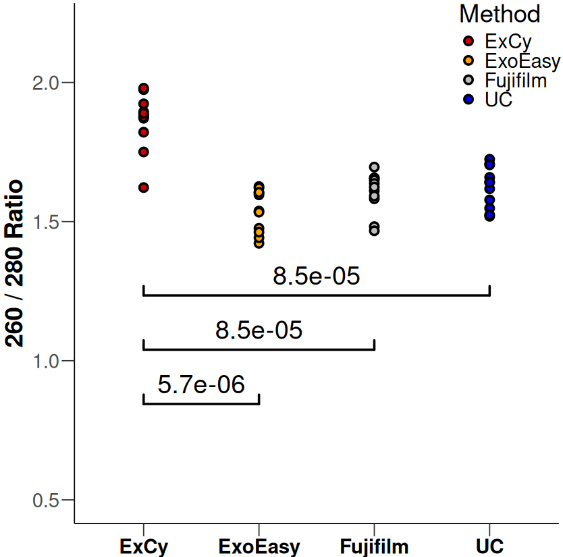


**Figure s6**. 260/280 nm^-1^ analysis to examine RNA purity after applying Qiagen’s miRNeasy kit to extract total RNA. One way Anova was performed with tukey’s post-hoc test and values were reported as comparison between ExCy and other methods


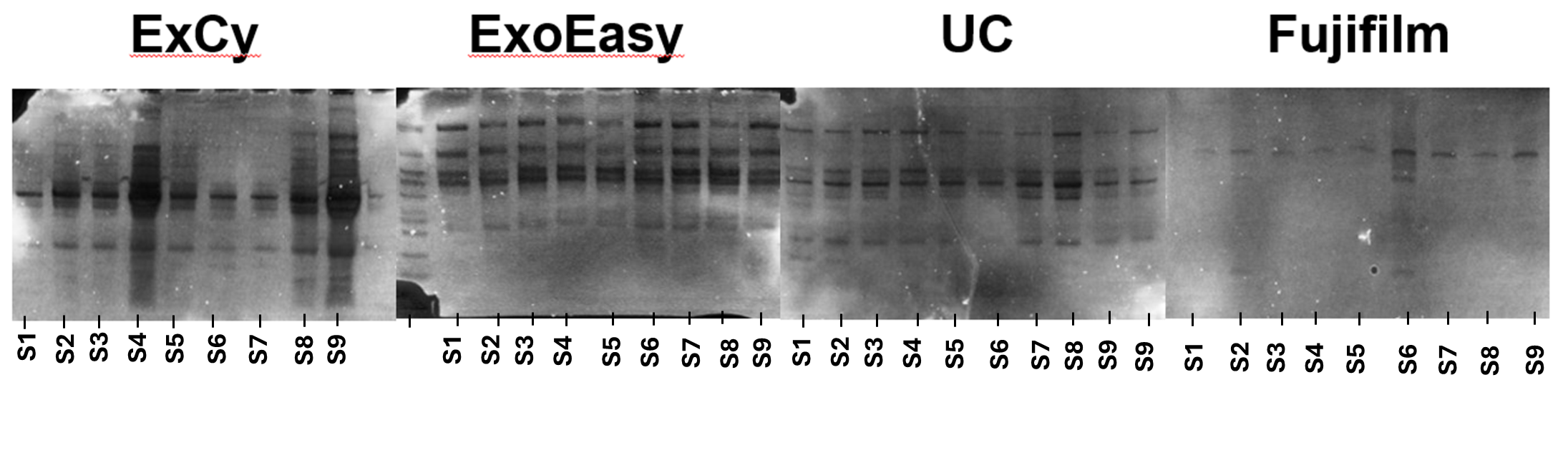


**Figure s7**. Protein gel analysis by Coomassie blue staining through BioRad imaging, within a tetra system, on the same sample for the four EV isolation methods. S9 was added twice to UC, out of random selection, to assess protein gel variation.


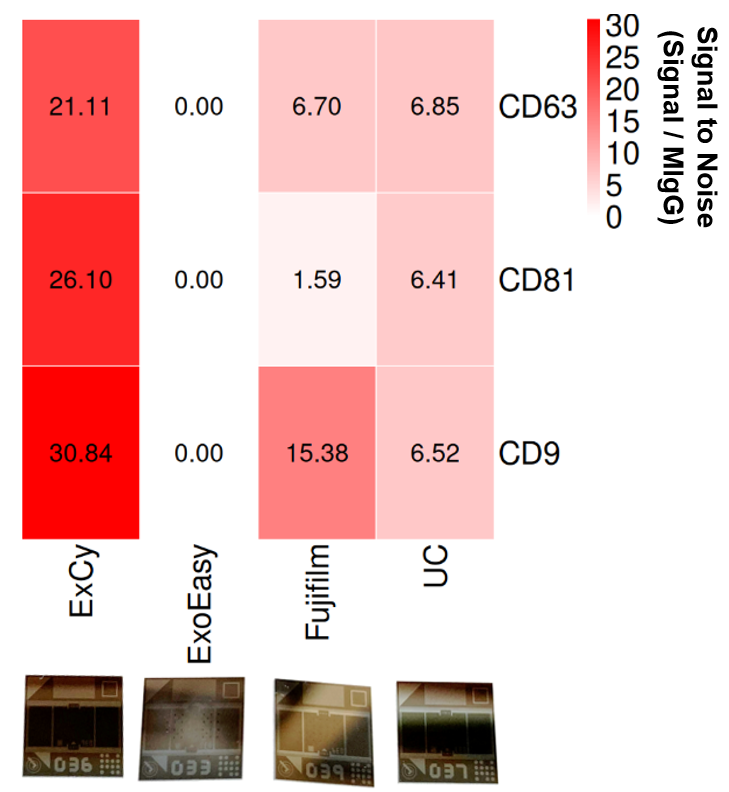


**Figure s8.** Heatmap depicting the ExoView analysis of particles captured and detected by CD63, CD81, CD9 relative to the background capture spot, mouse IgG (MIgG). ExoEasy was not analyzed due to massive precipitation and debris remaining even after significant washing steps.


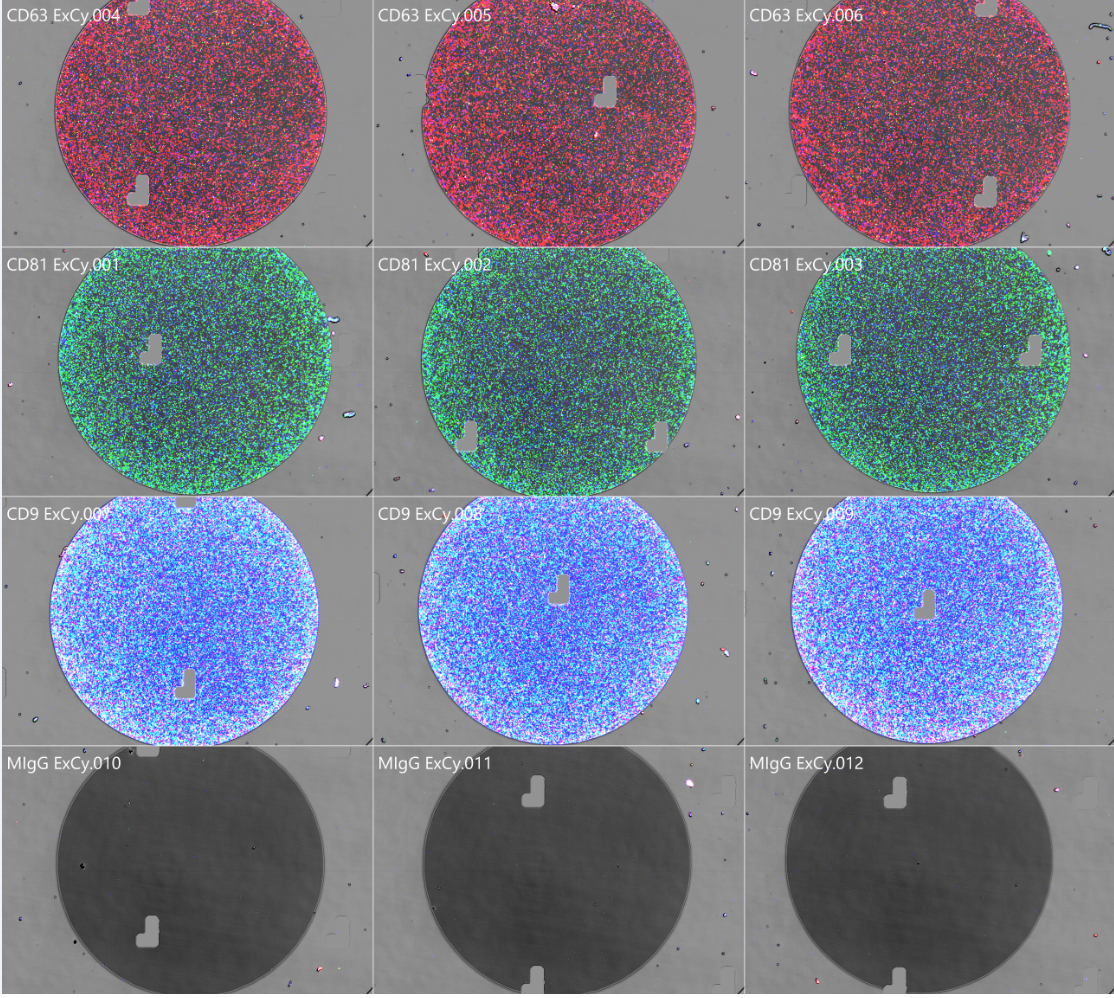


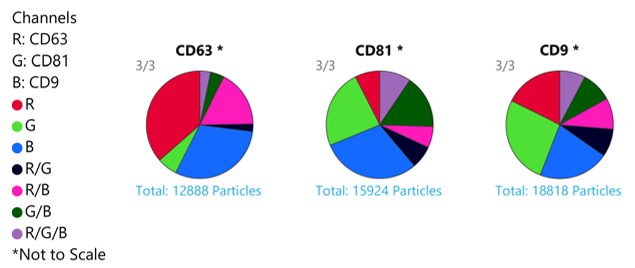


**Figure s9.** ExCy’s EV isolate tetraspanin (CD63, CD81, and CD9) co-localization analysis through the ExoView platform. Top. Single particle and fluorescent images regarding EVs captured and detected. Bottom. Pie charts analyzing the co-localized signatures for the particles isolated by ExCy.


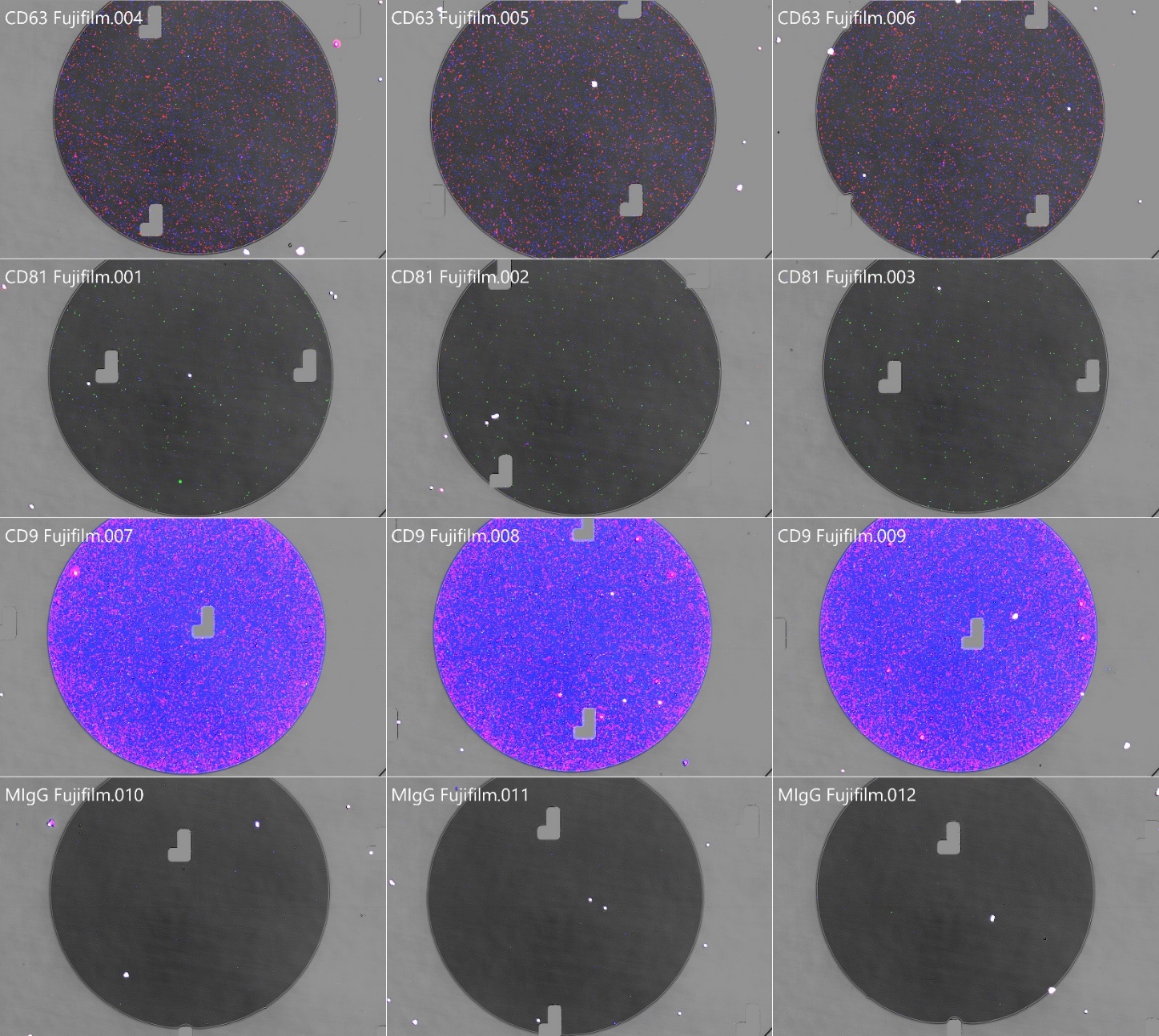


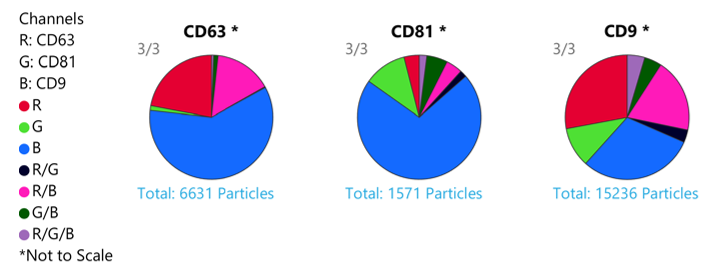


**Figure s10.** Fujifilm’s EV isolate tetraspanin (CD63, CD81, and CD9) co-localization analysis through the ExoView platform. Top. Single particle and fluorescent images regarding EVs captured and detected. Bottom. Pie charts analyzing the co-localized signatures for the particles isolated by Fujifilm


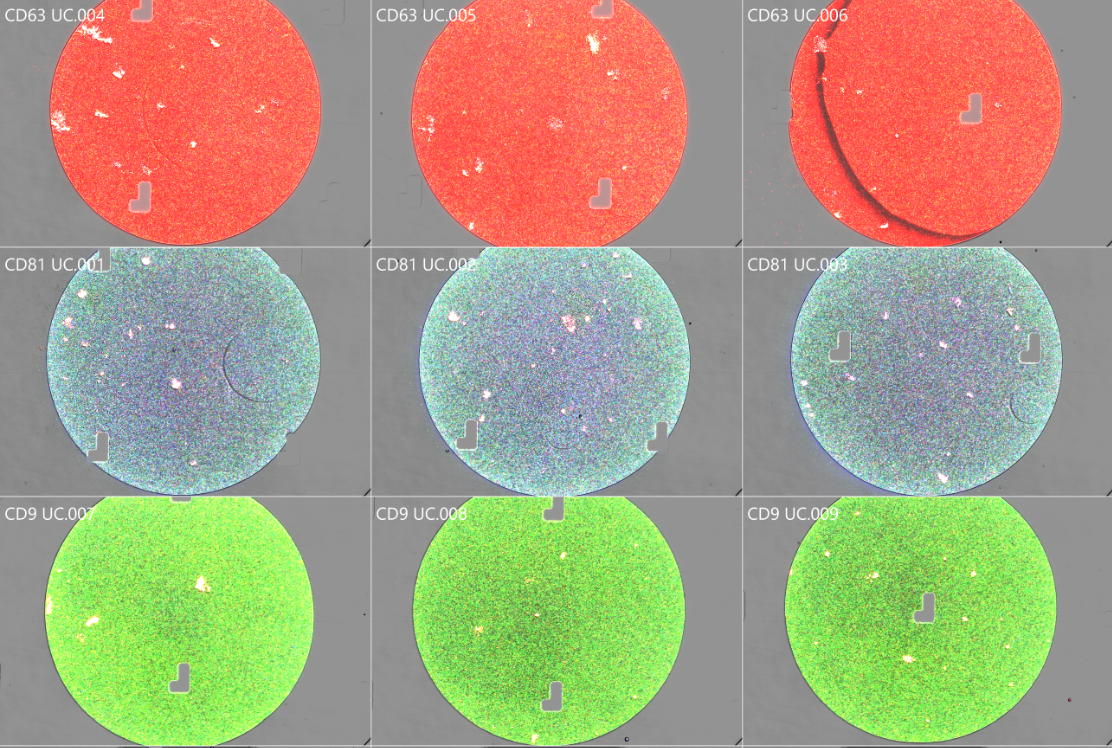


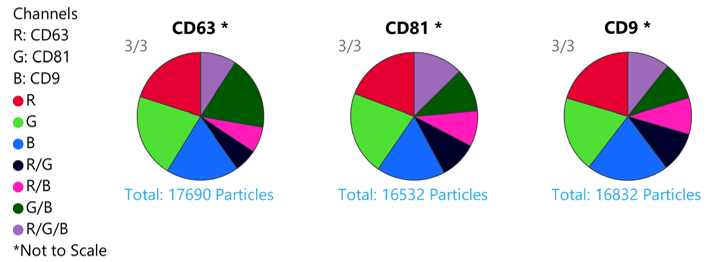


**Figure s11.** UC’s EV isolate tetraspanin (CD63, CD81, and CD9) co-localization analysis through the ExoView platform. Top. Single particle and fluorescent images regarding EVs captured and detected. Bottom. Pie charts analyzing the co-localized signatures for the particles isolated by UC.


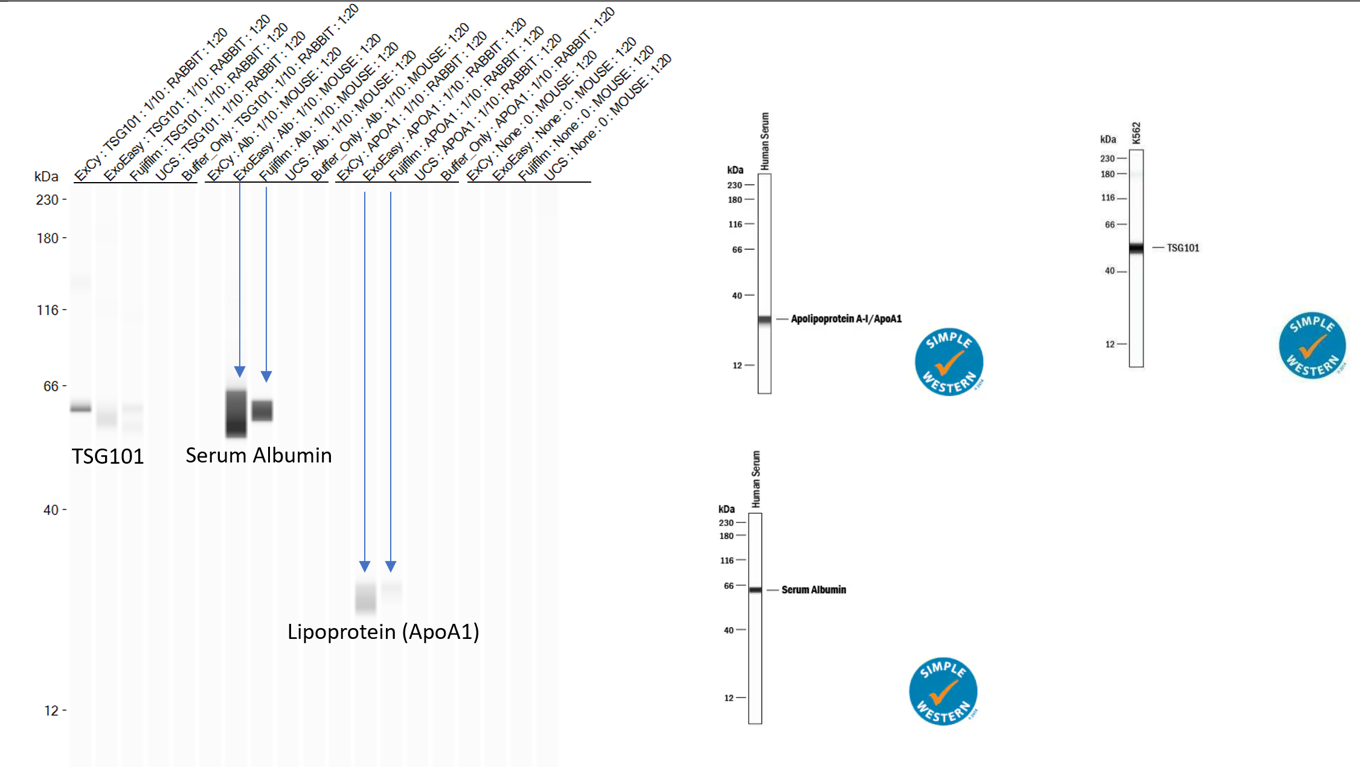


**Figure s12**. Western blot performed through the Simple Western assay to detect EV-associated biomarker and contamination from plasma. TSG101 is a highly conserved EV marker and indicator of present EVs. Serum Albumin and apolipoprotein-A1 (ApoA1) are highly abundant plasma proteins and frequent co-isolates in EV isolate samples. Blue arrows are shown to highlight the plasma protein co-isolate presence in ExoEasy and Fujifilm.


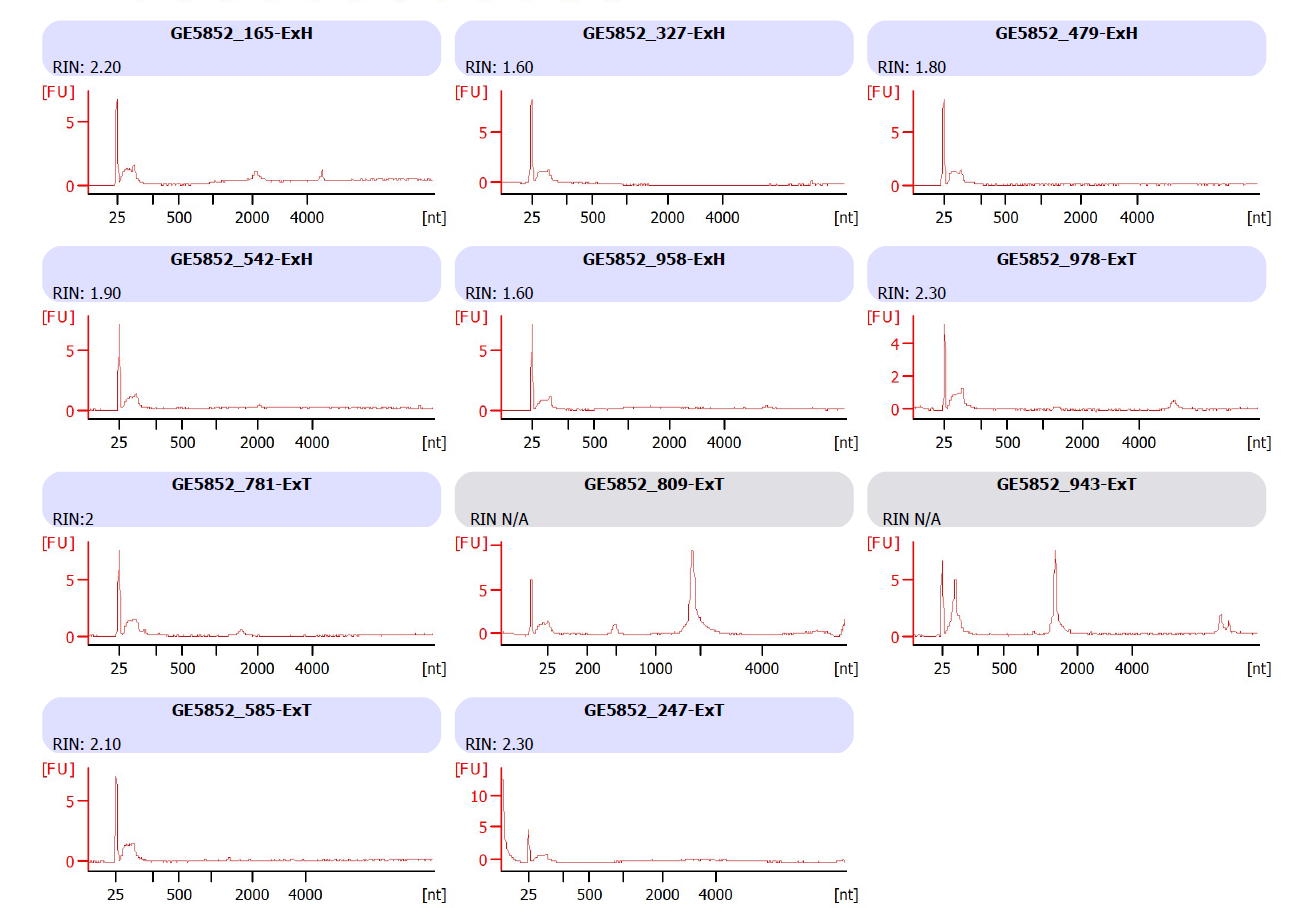


**Figure s13**. Bioanalyzer analysis on ExCy extracted RNA


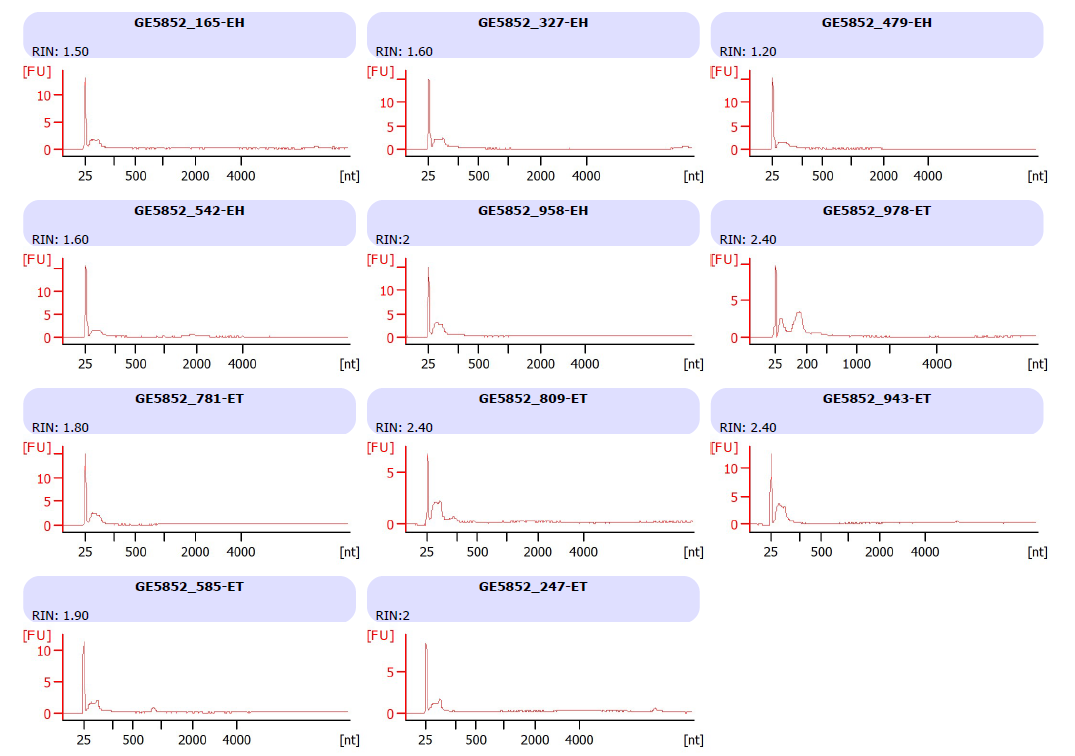


**Figure s14**. Bioanalyzer results on ExoEasy extracted RNA


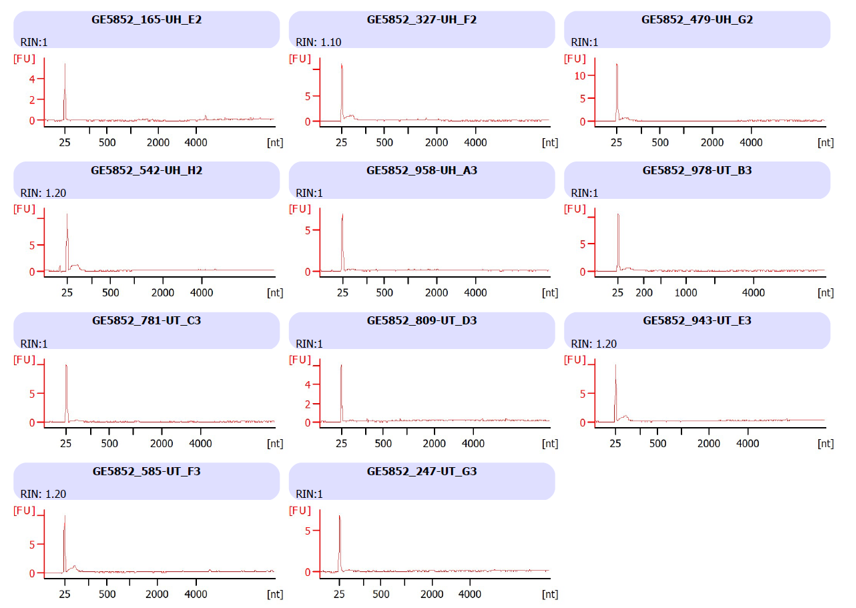


**Figure s15**. Bioanalyzer results on Ultracentrifugation extracted RNAs


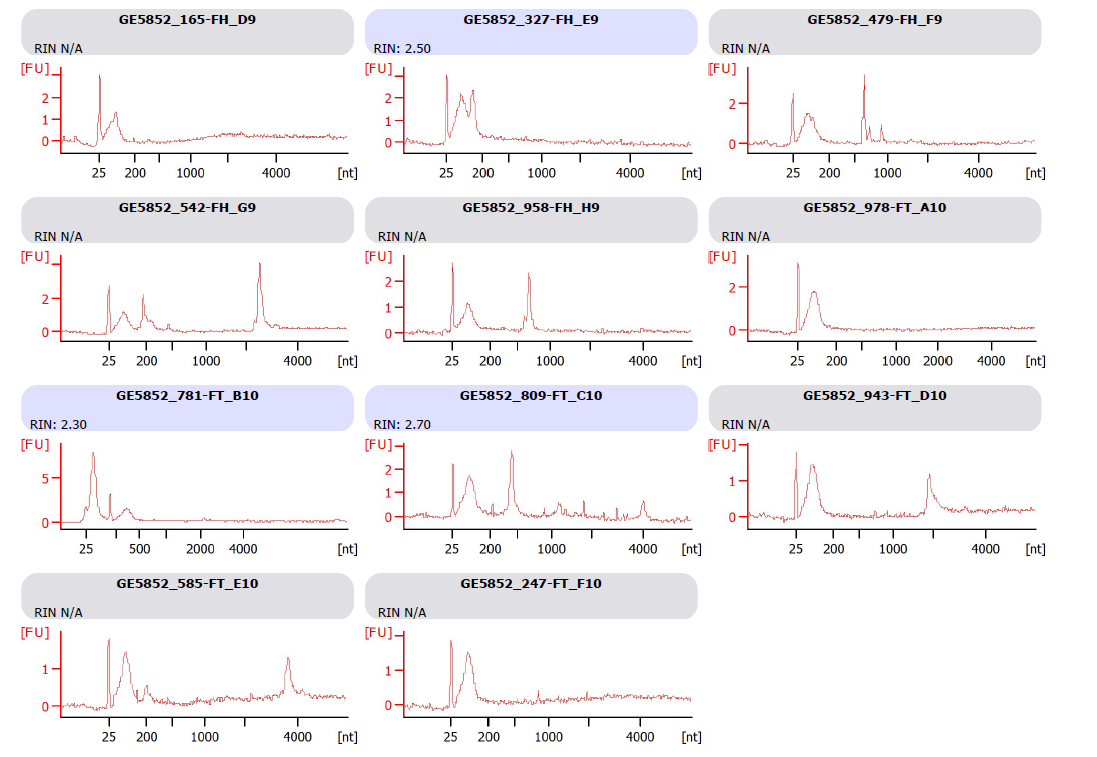
**Figure s16**. Bioanalyzer results on Fujifilm extracted RNAs

**Table s2.** HISAT2 mapping rate across each method for all the patients.

| **Patients** | **ExCy** | **ExoEasy** | **FujiFilm** | **UC** |
| --- | --- | --- | --- | --- |
| **PC1** | 75.98 | 71.06 | 68.91 | 61.67 |
| **H1** | 80.94 | 78.84 | 89.93 | 60.74 |
| **H2** | 86.38 | 81.31 | 77.37 | 71.15 |
| **PC2** | 79.21 | 57.3 | 60.04 | 57.89 |
| **PC3** | 73.36 | 57.38 | 67.28 | 61.93 |
| **H3** | 77.34 | 78.23 | 74.23 | 62.46 |
| **PC4** | 68.09 | 73.53 | 79.91 | 67.74 |
| **AVG ±** **STDEV:** | 77.33 ± 5.79 | 71.09 ±10.00 | 73.95 ± 9.71 | 63.37 ±4.50 |

**Table s3**. Total RNA distributions across each method for all patients

**ExCy**


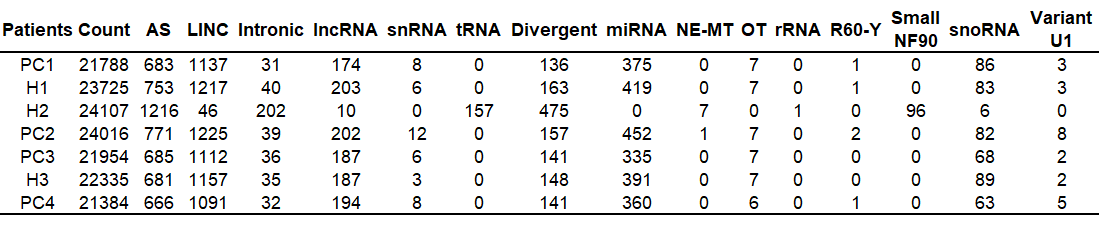


**ExoEasy**


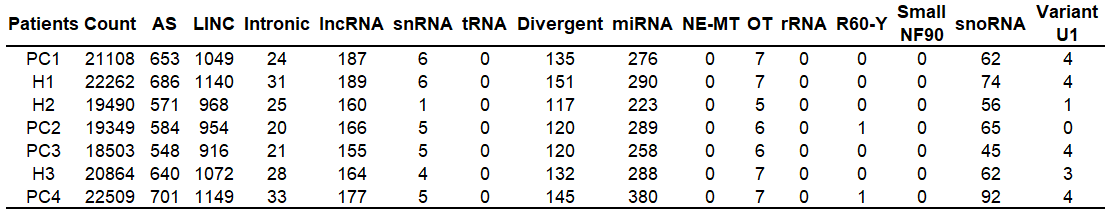


**Fujifilm**


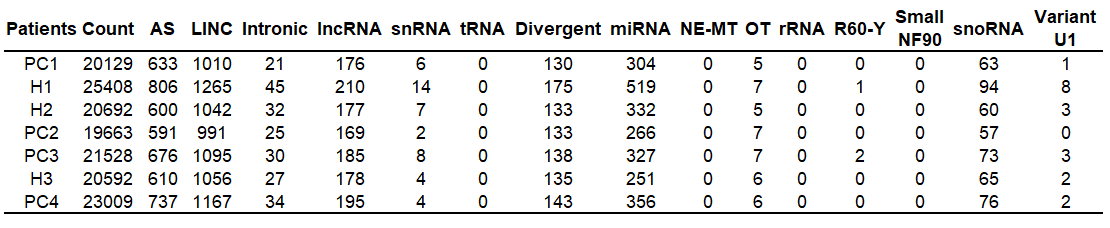


**UC**


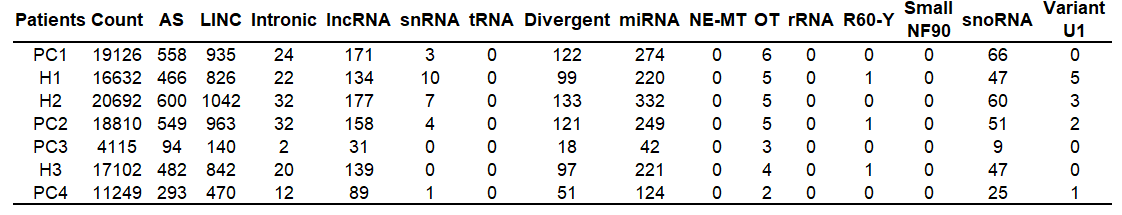


**Table s4**. Total RNA distributions across each method for all patients after mapping to Vesiclepedia.

**ExCy**


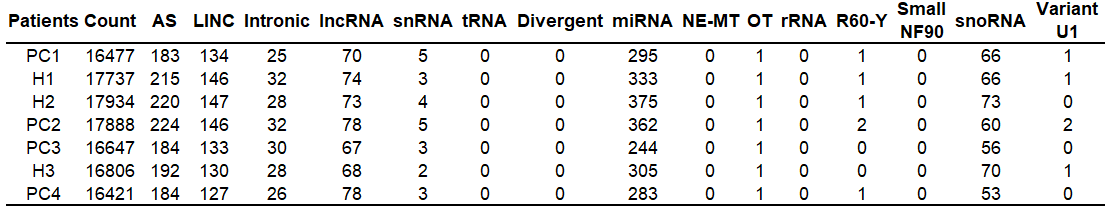


**ExoEasy**


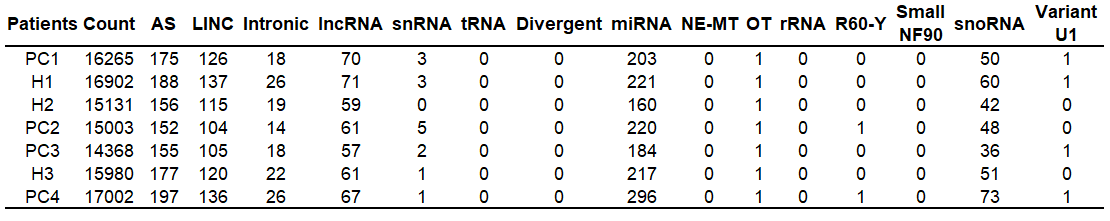


**Fujifilm**


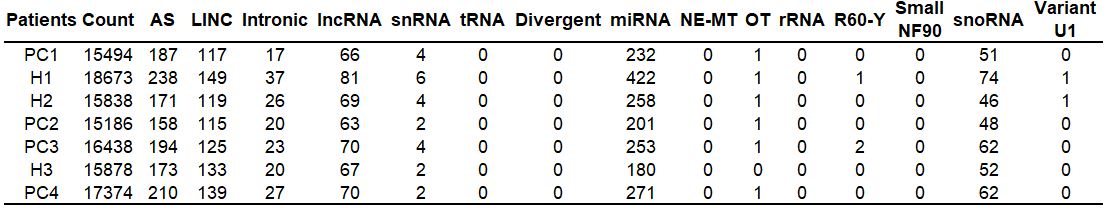


**UC**


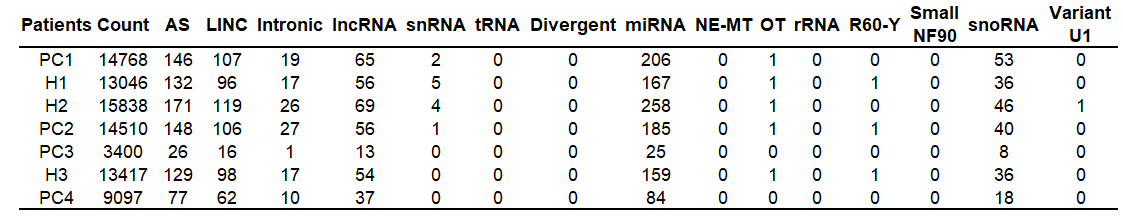


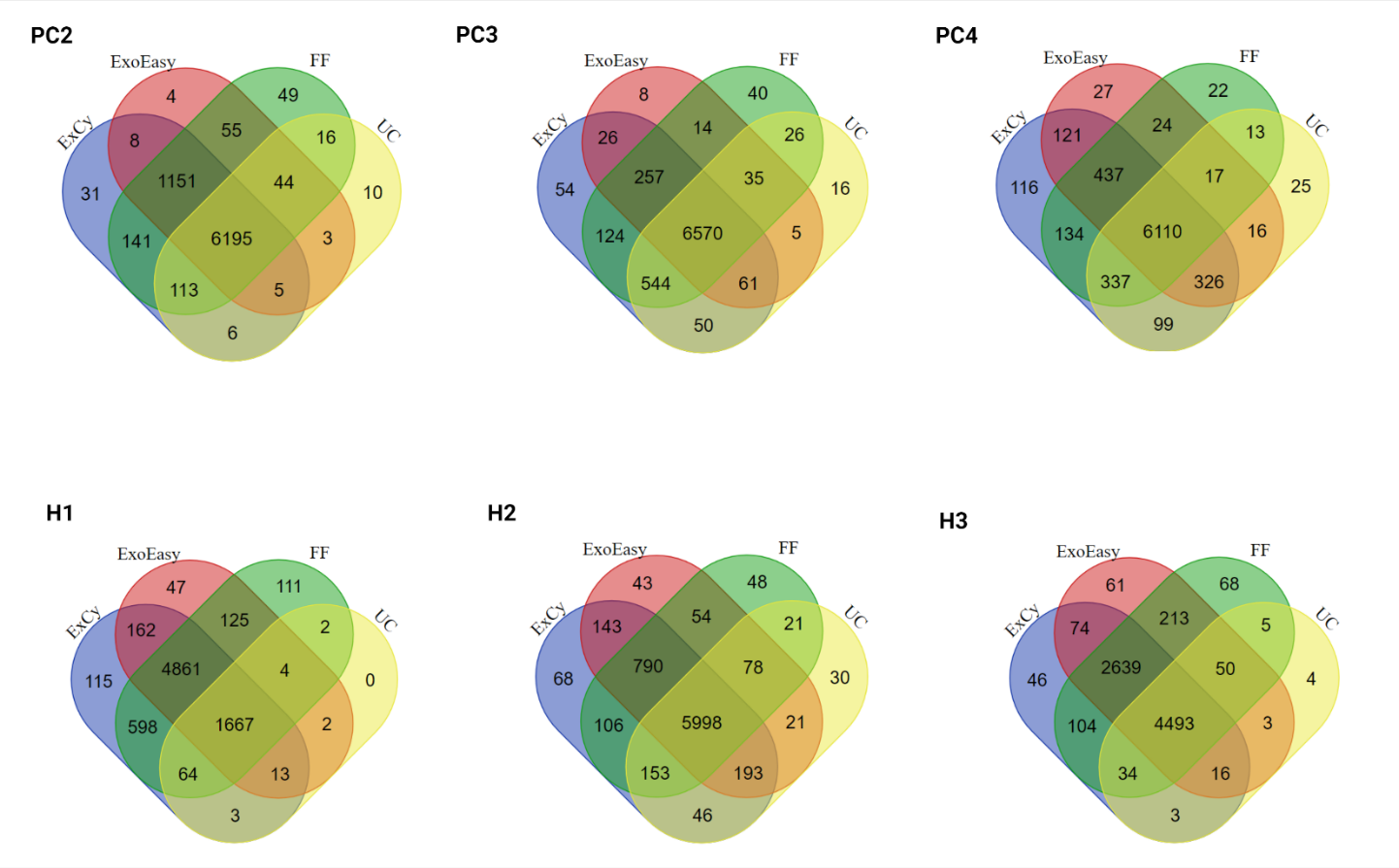


**Figure s17**. Venn diagrams indicating unique and shared mRNA transcripts across different methods for each patient.


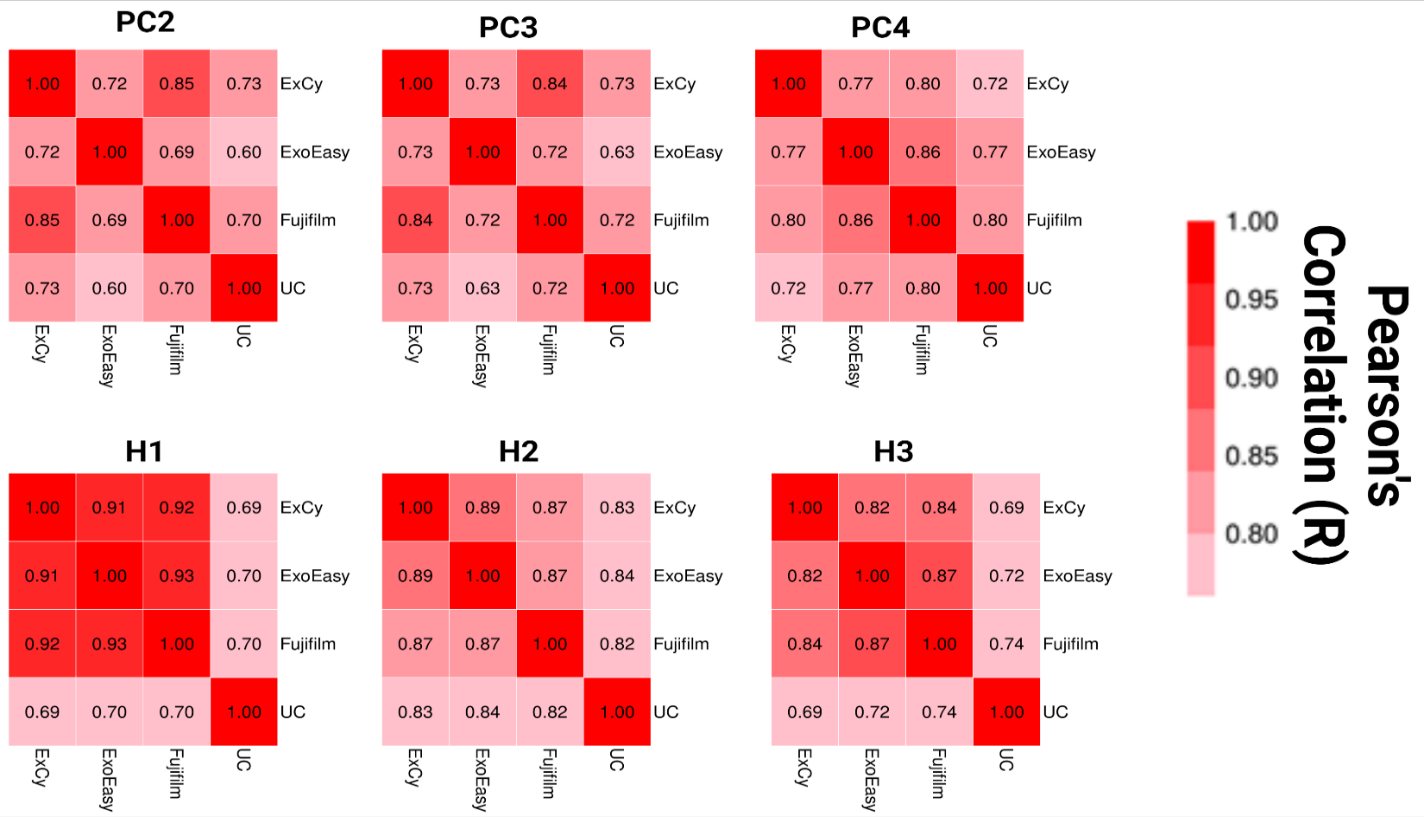


**Figure s18.** Pearson’s correlation matrix across each method for all patients.


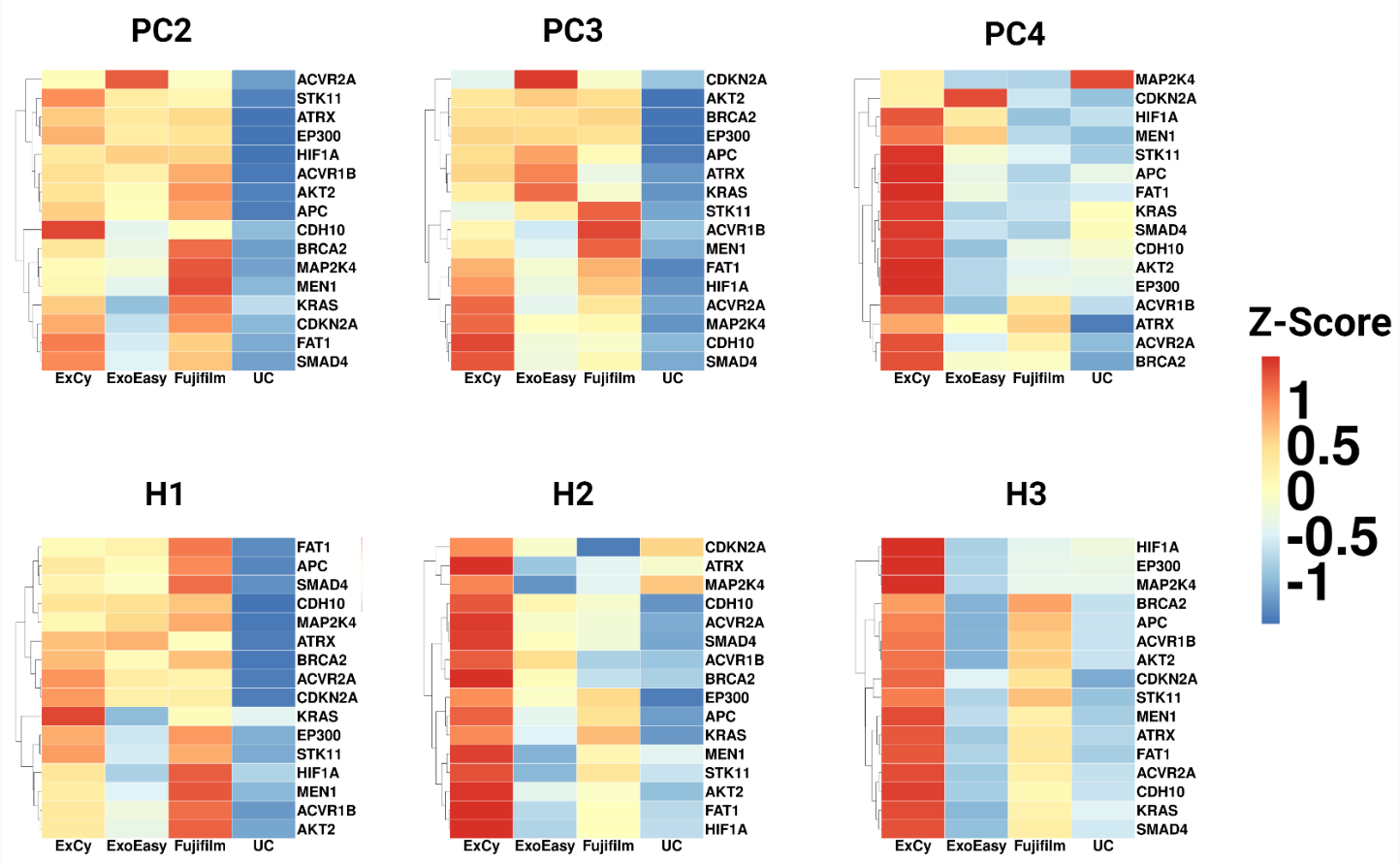


**Figure s19.** COSMIC pancreatic cancer heatmaps across all the other patients


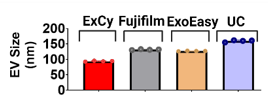


**Figure s20**. Summary of the EV sizes observed by transmission electron microscopy for figure 4.

**Table s4**. Investigating the differential analysis of EVs isolated by ExCy, ExoEasy, and Fujifilm from healthy samples to understand marker discovery differences with edgeR. **a)** ExCy was compared to ExoEasy and **b)** Fujifilm separately, then **c)** compared to the combined statistical effect of ExoEasy and Fujifilm. **d)** Fujifilm compared to ExoEasy.

**a**) **ExCy compared to ExoEasy**


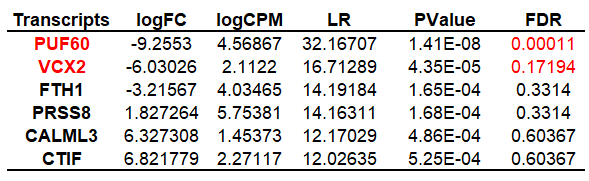


**b) ExCy compared to Fujifilm**


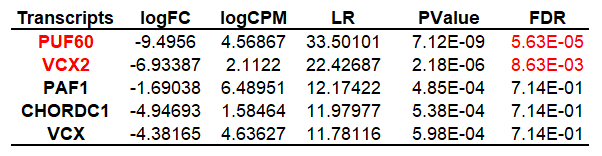


**c) ExCy compared to ExoEasy AND Fujifilm**


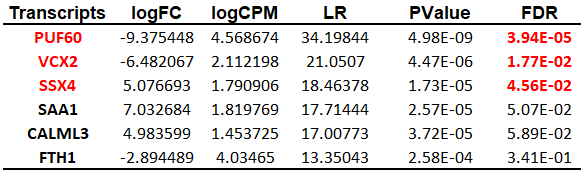


**d) Fujifilm compared to ExoEasy**


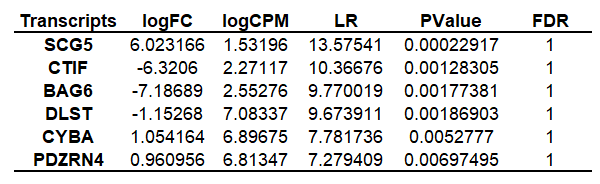


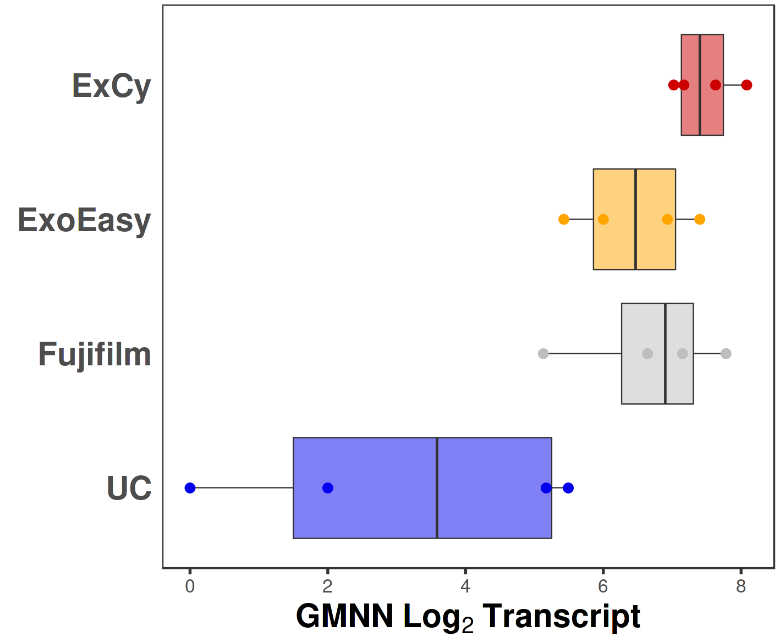


**Figure s21**. GMNN mRNA levels across the four EV isolation methods to understand interaction with PHC1. If PHC1 was detected in an EV, then GMNN must likely be isolated along with PHC1, since GMNN is regulated by PHC1 in the pancreas.


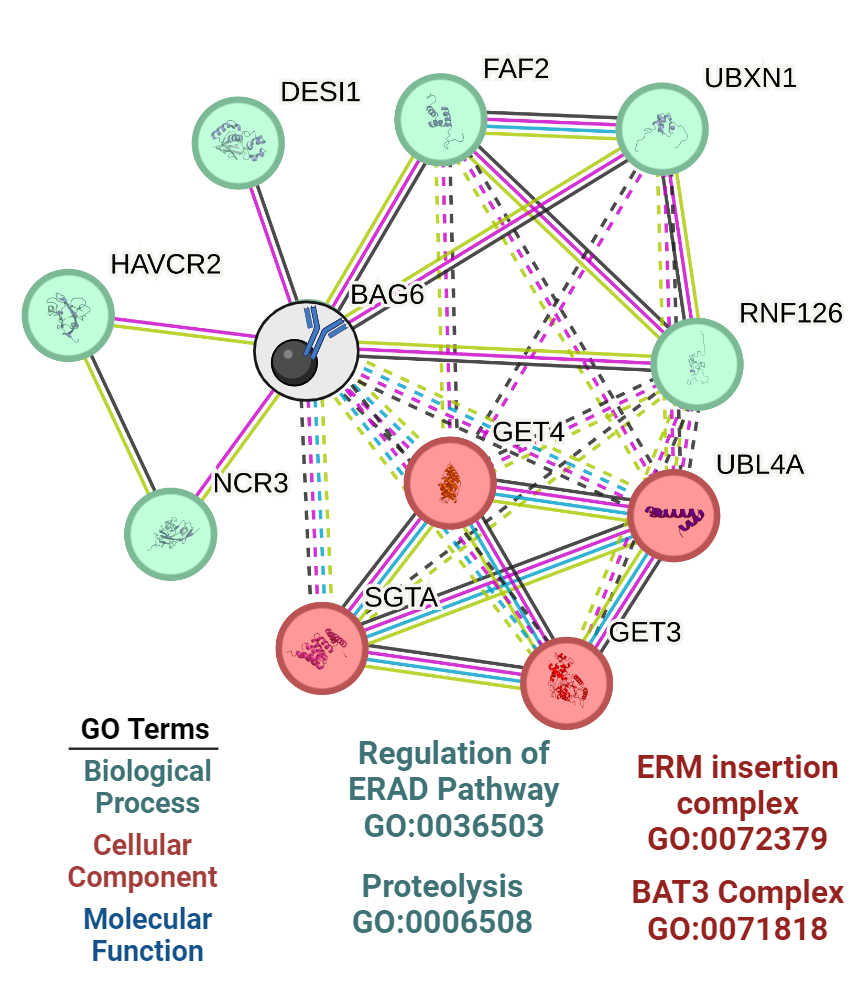


**Figure s22**. STRING analysis of Fujifilm’s differential pancreatic cancer marker, BAG6. The group colors represent k-means determined clustering of protein-to-protein interactions to show areas of strong local protein-protein interactions.


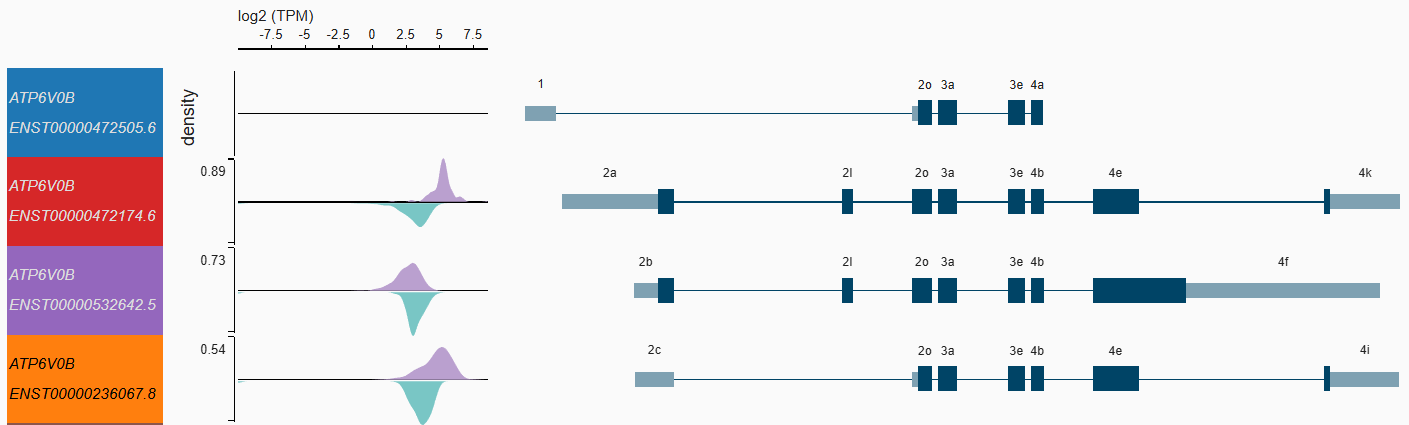


**Figure s23.** ATP6V0b’s differential exon analysis at the population level**,** comparing TCGA PDAC against GTEx through UCSC’s Xena pipeline.


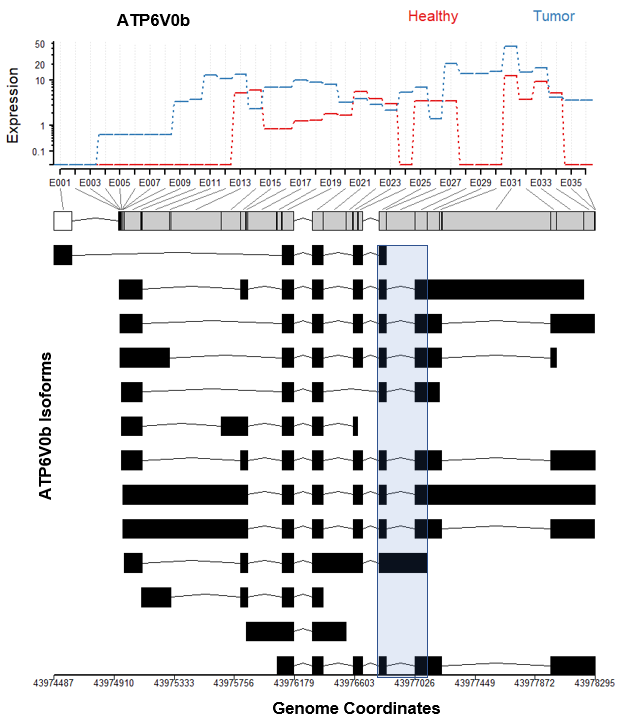


**Figure s24**. ATP6V0b’s differential exon analysis from the FREYA pipeline to locate the qPCR target region of interest. Genome coordinates refer to hg38. The blue shaded region indicates the region of interest used in ATP6V0b’s detection by qPCR.

**Table s5**. External patient cohort to validate ATP6V0b’s pancreatic cancer biomarker potential


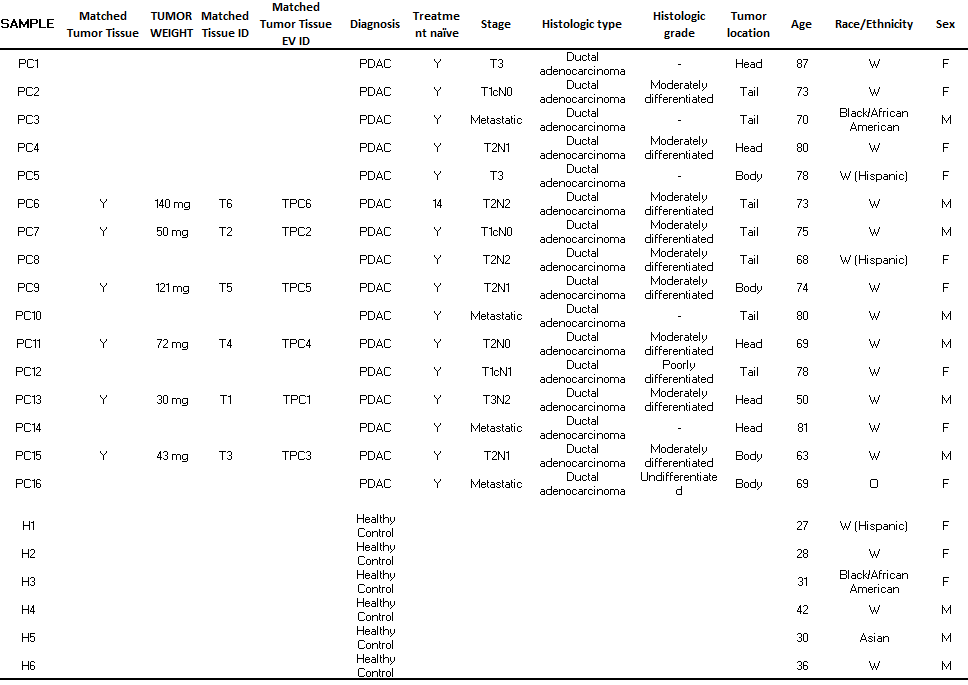


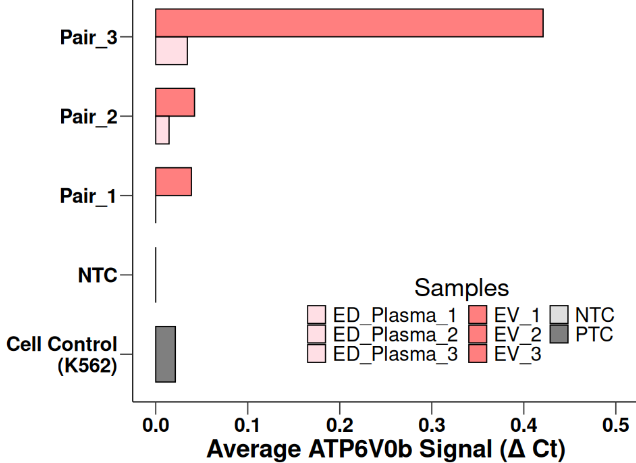


**Figure s25** – ATP6V0b detection by qPCR in plasma samples isolated by ExCy and compared to their EV depleted plasma counterpart. EEF1A1 was used as the normalizer for the **Δ Ct** calculation. The cell control was the K562 cell line, which served as our positive control template.

*
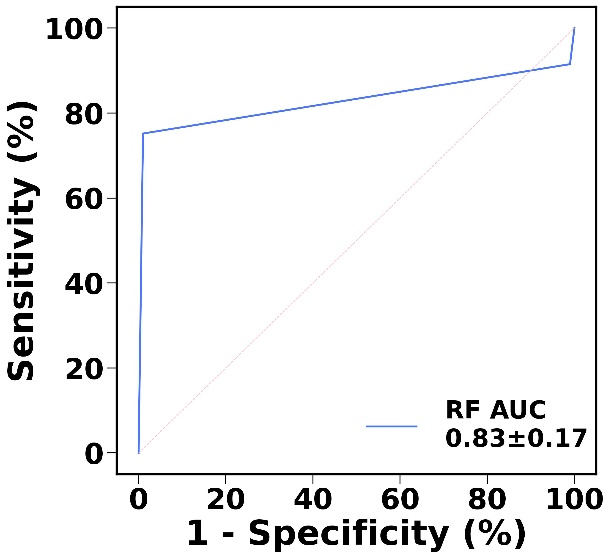
*

**Figure s26 –** ATP6V0b AUROC plot to classify metastatic vs non-metastatic cases using random forest.


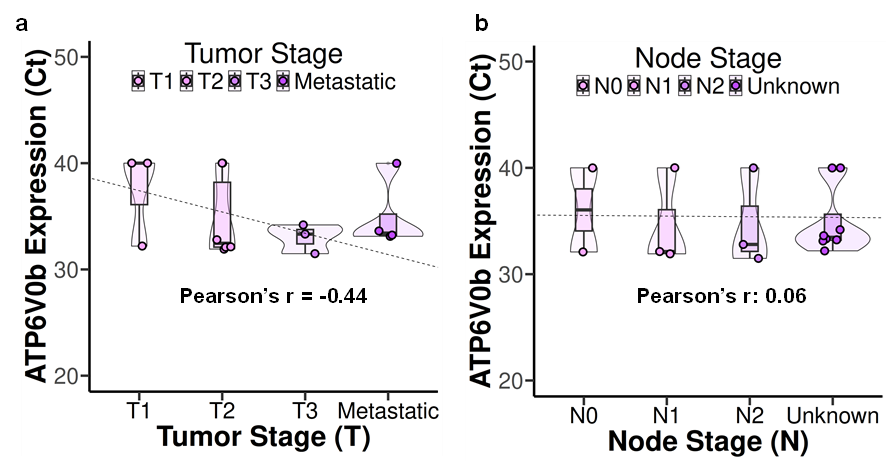


**Fig s27.** ATP6V0b expression correlation based on TNM staging. **Fig 27a**. ATP6V0b correlated to tumor stage (T). **Fig 27b**. ATP6V0b correlated to node stage (N)
