# Supplementary material for: Nanomaterial isolated extracellular vesicles enable high precision identification of tumor biomarkers for pancreatic cancer liquid biopsy
Source: J Nanobiotechnology. 2025 Jul 1;23:467. doi: 10.1186/s12951-025-03527-3 (PMC12211367; doi:10.1186/s12951-025-03527-3)
Supplement: Supplementary file 2 — Additional file 2. ExoQuality Index Dataset and Sequencing information. The dataset used to compute the EQI and sequencing information regarding HISAT2’s RNA annotations per sample across all methods before and after Vesiclepedia mapping. FastQC reports – External quality reports using FastQC, before and after applying trimmomatic, detailing each EV isolation’s transcriptomic sample assembly right after sequencing by the NovaSeq 6000. Each report will present basic assembly statistics followed by listing quality metrics including, Per base sequence quality, Per tile sequence quality, Per sequence quality, Per base sequence content, Per sequence GC content, Per base N content, Sequence Length Distribution, Sequence Duplication levels, Overrepresented sequences, and adaptor content. [file 12951_2025_3527_MOESM2_ESM.zip › FASTQC/FASTQC AFTER/Patient_11_ExCy__fastqc.html]

479-H\_S11\_L003\_R1\_001.fastq.gz FastQC Report 

FastQC Report

Sun 25 Jun 2023  
479-H\_S11\_L003\_R1\_001.fastq.gz

## Summary

- Basic Statistics
- Per base sequence quality
- Per tile sequence quality
- Per sequence quality scores
- Per base sequence content
- Per sequence GC content
- Per base N content
- Sequence Length Distribution
- Sequence Duplication Levels
- Overrepresented sequences
- Adapter Content

## Basic Statistics

| Measure | Value |
| --- | --- |
| Filename | 479-H\_S11\_L003\_R1\_001.fastq.gz |
| File type | Conventional base calls |
| Encoding | Sanger / Illumina 1.9 |
| Total Sequences | 79766711 |
| Sequences flagged as poor quality | 0 |
| Sequence length | 151 |
| %GC | 43 |

## Per base sequence quality

## Per tile sequence quality

## Per sequence quality scores

## Per base sequence content

## Per sequence GC content

## Per base N content

## Sequence Length Distribution

## Sequence Duplication Levels

## Overrepresented sequences

No overrepresented sequences

## Adapter Content

Produced by FastQC (version 0.11.7)
